# Telomere dysfunction activates YAP1 to drive tissue inflammation

**DOI:** 10.1038/s41467-020-18420-w

**Published:** 2020-09-21

**Authors:** Deepavali Chakravarti, Baoli Hu, Xizeng Mao, Asif Rashid, Jiexi Li, Jun Li, Wen-ting Liao, Elizabeth M. Whitley, Prasenjit Dey, Pingping Hou, Kyle A. LaBella, Andrew Chang, Guocan Wang, Denise J. Spring, Pingna Deng, Di Zhao, Xin Liang, Zhengdao Lan, Yiyun Lin, Sharmistha Sarkar, Christopher Terranova, Yonathan Lissanu Deribe, Sarah E. Blutt, Pablo Okhuysen, Jianhua Zhang, Eduardo Vilar, Ole Haagen Nielsen, Andrew Dupont, Mamoun Younes, Kalyani R. Patel, Noah F. Shroyer, Kunal Rai, Mary K. Estes, Y. Alan Wang, Alison A. Bertuch, Ronald A. DePinho

**Affiliations:** 1 Department of Cancer Biology, The University of Texas MD Anderson Cancer Center, Houston, TX 77030 USA; 2 Division of Pediatric Neurosurgery, Children’s Hospital of Pittsburgh of UPMC, Pittsburgh, PA 15224 USA; 3 Department of Neurological Surgery, University of Pittsburgh School of Medicine, Pittsburgh, PA 15261 USA; 4 Department of Genomic Medicine, The University of Texas MD Anderson Cancer Center, Houston, TX 77030 USA; 5 Department of Pathology, The University of Texas MD Anderson Cancer Center, Houston, TX 77030 USA; 6 Department of Pathology, School of Basic Medical Sciences, Southern Medical University, Guangzhou, China; 7 Department of Veterinary Medicine & Surgery, The University of Texas MD Anderson Cancer Center, Houston, TX 77030 USA; 8 Department of Immunology, Roswell Park Comprehensive Cancer Center, Buffalo, NY 14263 USA; 9 Department of Genitourinary Medical Oncology, The University of Texas MD Anderson Cancer Center, Houston, TX 77030 USA; 10 Division of Neurocritical Care, Department of Neurosurgery, Emory University, Atlanta, GA 30303 USA; 11 Department of Thoracic and Cardiovascular Surgery, The University of Texas MD Anderson Cancer Center, Houston, TX 77030 USA; 12 Department of Molecular Virology and Microbiology, Baylor College of Medicine, Houston, TX 77030 USA; 13 Department of Infectious Diseases, The University of Texas MD Anderson Cancer Center, Houston, TX 77030 USA; 14 Department of Clinical Cancer Prevention, The University of Texas MD Anderson Cancer Center, Houston, TX 77030 USA; 15 Department of Gastroenterology, Herlev Hospital, University of Copenhagen, Herlev, DK-2730 Denmark; 16 Division of Gastroenterology, Hepatology and Nutrition, Department of Internal Medicine, McGovern Medical School, The University of Texas Health Science Center at Houston, Houston, TX 77030 USA; 17 Department of Pathology and Laboratory Medicine, University of Texas Health Science Center at Houston, McGovern Medical School and Memorial Hermann Hospital-TMC, Houston, TX 77030 USA; 18 Department of Pathology, Texas Children’s Hospital, Houston, TX 77030 USA; 19 Department of Medicine, Section of Gastroenterology and Hepatology, Baylor College of Medicine, Houston, TX 77030 USA; 20 Division of Hematology/Oncology, Department of Pediatrics, Baylor College of Medicine, Houston, TX 77030 USA; 21 Department of Molecular & Human Genetics, Baylor College of Medicine, Houston, TX 77030 USA

**Keywords:** Cancer genetics, Gastrointestinal diseases

## Abstract

Germline telomere maintenance defects are associated with an increased incidence of inflammatory diseases in humans, yet whether and how telomere dysfunction causes inflammation are not known. Here, we show that telomere dysfunction drives pATM/c-ABL-mediated activation of the YAP1 transcription factor, up-regulating the major pro-inflammatory factor, pro-IL-18. The colonic microbiome stimulates cytosolic receptors activating caspase-1 which cleaves pro-IL-18 into mature IL-18, leading to recruitment of interferon (IFN)-γ-secreting T cells and intestinal inflammation. Correspondingly, patients with germline telomere maintenance defects exhibit DNA damage (γH2AX) signaling together with elevated YAP1 and IL-18 expression. In mice with telomere dysfunction, telomerase reactivation in the intestinal epithelium or pharmacological inhibition of ATM, YAP1, or caspase-1 as well as antibiotic treatment, dramatically reduces IL-18 and intestinal inflammation. Thus, telomere dysfunction-induced activation of the ATM-YAP1-pro-IL-18 pathway in epithelium is a key instigator of tissue inflammation.

## Abstract (web summary)

How telomere dysfunction is directly linked to inflammation in humans is currently unclear. Here the authors reveal that telomere dysfunction drives activation of the YAP1 transcription factor, up-regulating the pro inflammatory factor, pro-IL-18 thus revealing a link between telomere dysfunction and initiation of intestinal inflammation.

## Introduction

Telomeres are repetitive nucleic acid and chromatin structures that cap chromosome ends and are maintained by telomerase, which consists of the catalytic telomerase reverse transcriptase (*TERT*) and RNA component (*TERC*)^1,2^. While telomere maintenance defects are linked to increased risk of chronic inflammatory conditions in humans^3–5^, whether telomere dysfunction directly drives inflammation or results from inflammation associated ROS remain unanswered questions. In *mTerc*-null mice, telomere dysfunction promotes liver inflammation and cirrhosis in the setting of CCL4-induced liver injury^6^. Correspondingly, humans with germline mutations in *TERC* or *TERT* can develop idiopathic pulmonary fibrosis; and chronic hepatocyte turnover with viral hepatitis can lead to liver inflammation and cirrhosis^4,7,8^. Humans with germline mutations in telomerase components show progressive villous atrophy, enterocolitis and intraepithelial lymphocytosis^7^. Patients with inflammatory bowel disease (IBD) (of which ulcerative colitis and Crohn’s disease are the two main entities) show increased telomere attrition in the intestinal epithelial cells which may even drive increased incidence of colon cancers^9–11^. A mechanistic understanding of how telomere dysfunction may contribute directly to intestinal inflammation and hence provide clues for increased incidence of other inflammatory conditions associated with telomere compromise has remained elusive. Recent groundbreaking mechanistic work by Karlseder and colleagues demonstrates that critically short telomeres in cellular crisis can activate a response mediated by the cGAS/STING pathway leading to massive cell death mediated through autophagy, indicating that telomere dysfunction may play a role to initiate inflammation^12^.

Here, we uncovered an intestinal inflammatory phenotype in mice that have telomere dysfunction, predominantly involving colon in a segmental pattern. These pathologies prompted us to explore whether telomere dysfunction itself can serve as a primary initiator and driver of inflammation, revealing a previously un-identified pro-inflammatory pathway in mice and humans.

## Results

### Telomere dysfunction in the gut epithelia drives inflammation

To study telomere biology in the intestine, we generated mice harboring (i) a tamoxifen (TAM)-inducible Cre allele driven by the Lgr5 promoter (Lgr5-GFP-IRES-CreER^T2^) that directs GFP and CreER^T2^ expression to Lgr5+ epithelium, and (ii) an *mTert* allele that can be reactivated by Cre-mediated deletion of a transcriptional stopper cassette flanked by LoxP sites (LSL-mTert). Intergenerational crosses of homozygous LSL-mTert mice result in progressive telomere loss with onset of DNA damage signaling by generations 3 (G3) and 4 (G4), causing tissue stem cell depletion, organ atrophy and premature aging^13^.

**Fig. 1:**
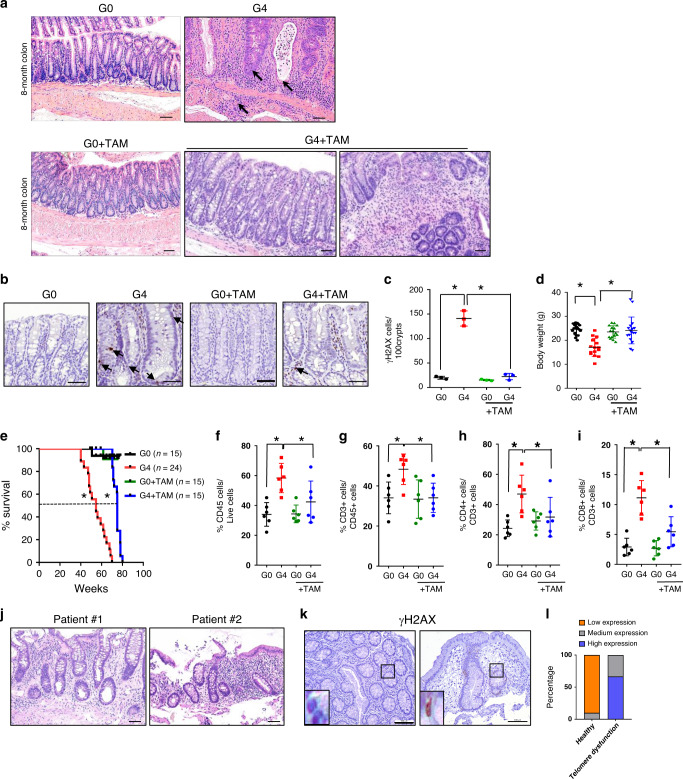
Telomere dysfunction in the gut epithelia drives inflammation. **a** Hematoxylin and eosin staining of colon tissue from 8-month old G0 and G4 mice treated with and without tamoxifen (*n* = 15), scale bar 100 µm. Arrows indicate cryptitis, crypt abscess and submucosal inflammation. **b** Immunohistochemical staining for γH2AX (arrows) in the colon of mice at 8-month old G0 and G4 mice treated with and without tamoxifen (*n* = 3), scale bar 50 µm. **c** Quantification of the γH2AX stained cells in 100 colonic crypts from G0 and G4 mice treated with and without tamoxifen (*n* = 3). *P* values were calculated using two sided *t* test between G0 and G4 (*p* < 0.0002), between G4 and G4 + TAM (*p* < 0.0003). **d** Change in body weight (g) at time of death of telomere proficient G0 and telomere deficient G4 mice treated with and without tamoxifen (G0, *n* = 18, G4, *n* = 15, G0 = TAM, *n* = 16, G4 + TAM, *n* = 17). *P* values were calculated using two sided t test between G0 and G4 (*p* = <0.0001), between G4 and G4 + TAM (*p* < 0.0003). **e** Survival curves of mice of the indicated genotypes treated with or without tamoxifen. Dotted line indicates 50% survival of G4 mice (54.6 weeks) and G4 + TAM mice (75 weeks) (G0 mice, *n* = 15; G4, *n* = 24; G0 + TAM, *n* = 15; G4 + TAM, *n* = 15). *P* values were calculated using Mantel-cox test, between G0 and G4 (*p* < 0.0001) and between G4 and G4 + TAM (*p* < 0.0001). **f**–**i** Percentage of lamina propria and intestinal epithelial immune cells present in the colon of the mice of the indicated genotypes treated with or without tamoxifen (*n* = 6). *P* values were calculated using two sided *t* test between G0 and G4, G4 and G4 + TAM. **f** CD45, *p* value between G0 and G4, *p* = 0.0008, between G4 and G4 = TAM, *p* = 0.0451. **g** CD3, *p* value between G0 and G4, *p* = 0.0097, between G4 and G4 + TAM, *p* = 0.0079. **h** CD4, *p* value between G0 and G4, *p* = 0.0022, between G4 and G4 + TAM, *p* = 0.0551. **i** CD8, *p* value between G0 and G4, *p* < 0.0001, between G4 and G4 + TAM, *p* = 0.0045. **j** Hematoxylin and eosin staining of colonic biopsies from patients with mutations in TERT or proteins of the shelterin complex, TIN2 (*n* = 3). Colonic biopsies from two patients show cryptitis, increased apoptosis, increased chronic inflammation in the lamina propria, and mild crypt distortion. **k** Immunohistochemistry with the colonic epithelium from healthy pediatric patients/(controls) and patients with telomeropathies for γH2AX (Control, *n* = 10; telomere dysfunctional patients, *n* = 3). **l** Histogram depicting the quantification of staining intensity for γH2AX in the colonic epithelium of healthy (control) and telomere dysfunctional patients. γH2AX, *p* = 0.0001. *P* values were calculated using Fisher’s exact test. *statistically significant, *p* < 0.05 by unpaired Student’s *t* test, two tailed. *n*, number of mice or patient biopsies used in the study. Data are represented as mean ± SEM. Experiments were conducted at least two independent times. Also refer to Supplementary Fig. 1, Supplementary Fig. 2, and Supplementary Fig. 3 and Supplementary Table 1 and 2.

Serial histological analysis of the intestine was performed in G4 mice and telomerase active controls (G0). At 3 months of age (termed young), approximately 40% of G4 mice exhibited mild colonic degeneration (Supplementary Fig. 1a, c; Supplementary Table 1; see “Methods” for histological score definitions), exhibiting cryptitis and crypt abscesses, slight inflammation, and DNA damage (Supplementary Table 1; Supplementary Fig. 1a and 2g), while the G4 terminal ileum exhibited more modest pathology with mild cryptitis (Supplementary Fig. 1d). By 8 months of age (termed old), G0 intestines continued to exhibit minimal pathology, while all G4 colons developed multiple erosions and ulcerations along with significant cryptitis and crypt abscesses, with chronic inflammation, (Fig. 1a; Supplementary Table 1; Supplementary Fig. 1f), increased DNA damage in the intestinal epithelium (Fig. 1b, c, Supplementary Fig. 2d, e) and significant loss of body weight (Fig. 1d). The G4 terminal ileum showed modest pathology and the duodenum and jejunum were normal (Supplementary Fig. 1e). Immune profiles of the 8-month old G4 intestines showed abundant CD4^+^ and CD8^+^ T cells, B cells, macrophages and dendritic cells (Fig. 1f–i; Supplementary Fig. 2h–k). This considerable T cell infiltration gains significance in light of previous work establishing T cells as key drivers of inflammatory pathology^14,15^. Pathological analysis of young G4 mice with healthy intact intestines (low histological scores) already showed higher recruitment of CD4^+^ and CD8^+^ T cells in the lamina propria, despite overall lower lymphocyte count (CD45+ cells) relative to normal immune profiles of age-matched G0 mice (Supplementary Fig. 2l–o). Thus, we hypothesized that intrinsic DNA damage signaling emanating from G4 epithelium may secrete factors that recruit T cells to initiate tissue inflammation.

Given the systemic impact of telomere dysfunction across all organ systems, we sought to determine whether telomere dysfunction specifically in intestinal epithelia serves a direct role in maintaining the inflammatory pathology. To that end, *Tert* gene expression was reactivated only in Lgr5^+^ cells by TAM treatment in 1- and 6-month old G4 mice (G4 + TAM). The G0 + TAM mice with normal telomere function served as TAM treatment controls. Following TAM treatment, Cre-mediated LSL excision activated *Tert* expression and suppressed γH2AX foci in intestinal epithelia, consistent with telomerase reactivation and restoration of telomeres (Fig. 1b, c old mice; Supplementary Fig. 2b, f, g young mice). The older G4 + TAM mice presented with a gradient of rescue in their pathology, with partial rescue of cryptitis, crypt abscess and inflammation seen in 50% of the mice treated with tamoxifen and analyzed post 30 days (Supplementary Fig. 1g, i), and a striking rescue of severe pathologies of older G4 + TAM mice treated with TAM and analyzed post 60 days, with only 33% of these mice exhibiting epithelial hyperplasia, cryptitis, crypt abscess and moderate to severe inflammation (Fig. 1a; Supplementary Fig. 1g–i; Supplementary Table 1). At this timepoint, analysis of the intestines from telomerase reactivated mice revealed a significant clonal expansion of the Lgr5-EGFP + crypts (Supplementary Fig. 2a–c) indicative of intestinal regeneration. Analysis of DNA damage in the crypts identified a significant reduction in the number of γH2AX-positive Lgr5-EGFP+ cells in crypts that were reactivated for telomerase, suggesting an amelioration of damage in the stem cell compartment (Supplementary Fig. 2d, e). Consistent with disease resolution (Supplementary Table 1), older G4 + TAM mice showed restoration of normal body weight and increased overall survival relative to G4 controls (median survival increased by 20.4 weeks in G4 + TAM mice) (Fig. 1d, e). Flow cytometric analysis of ‘old’ and ‘young’ G4 + TAM intestines showed dramatic reductions in T cells (Fig. 1f–i; Supplementary Fig. 2l–o) as well as reductions in B cells, macrophages and dendritic cells (Supplementary Fig. 2i–k). We conclude that telomere dysfunction in intestinal epithelium initiates and maintains an inflammatory condition that can be reversed by restoration of telomere function.

To substantiate the telomere-inflammation link in humans, we analyzed a patient cohort of 21 patients from the Texas Children’s Hospital patient registry with telomere dysfunction presenting in childhood (median age 12 years at last follow up or death) of which intestinal biopsies were available for 8 patients who presented with gastrointestinal symptoms. These observations align with the report of increased gastrointestinal symptoms in the Hopkin’s telomere registry^7^. Signs of intestinal inflammation were observed in 5 of 8 patients (Fig. 1j; Supplementary Table 2), including acute enteritis/colitis with eosinophils and evidence of chronic inflammation in the lamina propria, increased crypt apoptosis, cryptitis, focal gland injury, mild lymphoplasmacytic inflammation with mildly regenerative crypt and significant intraepithelial T cell infiltrate (Fig. 1j). Elevated DNA damage signaling (γH2AX) was detected in these patient samples compared to age-matched normal controls (Fig. 1k, l). Together, these data are consistent with a driver role of telomere dysfunction in inflammation.

### Identification of YAP1 as a key regulator of immune pathways including *IL-18*

**Fig. 2:**
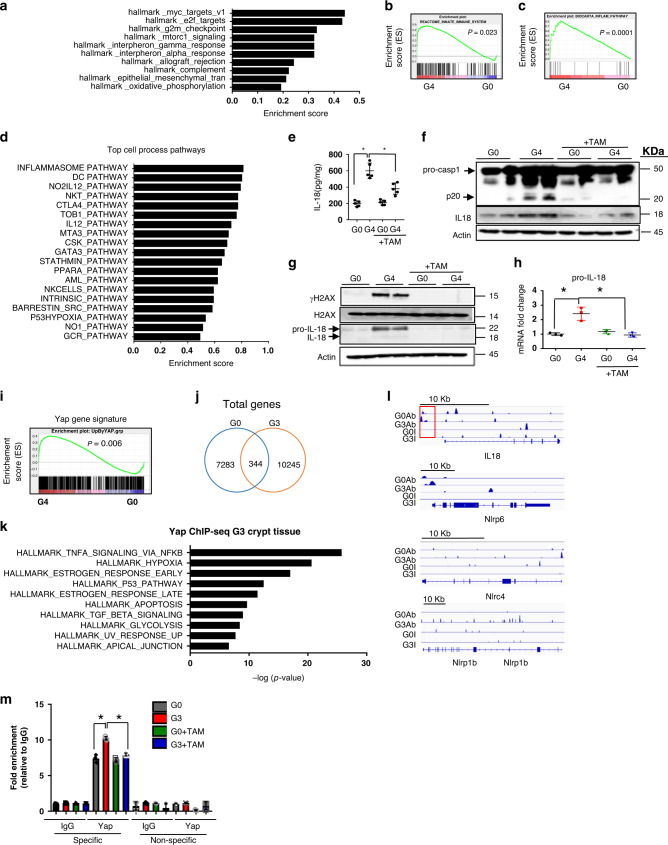
Identification of YAP1 as a key regulator of immune pathways including IL-18. **a** RNA-seq analysis and pathway analysis of colonic crypts from G0 and G4 mice identified several inflammation-related pathways (*n* = 2). **b** Gene set enrichment analysis curve from the RNA-seq analysis in panel (**a**) depicting the innate immune system pathway with a significant *p* value of 0.02. (*n* = 2). **c** Gene set enrichment analysis curve depicting the inflammasome pathway that was identified as the topmost deregulated pathway by RNA-seq from the GFP^+^ sorted colonic epithelial cells of G0 and G4 mice (*n* = 3). **d** Top cell process pathways identified by GSEA analysis from the GFP^+^ colonic sorted cells of G0 and G4 mice. (*n* = 2). **e** ELISA for IL-18 with the colonic lysates from the mice of the indicated genotypes (*n* = 5). *P* values were calculated using two sided *t* test between G0 and G4, G4 and G4 + TAM. *P* value between G0 and G4, *p* < 0.0001, between G4 and G4 + TAM, *p* = 0.0031. **f** Immunoblotting with caspase-1 and IL18 antibody of colonic epithelial lysates from G0 and G4 mice treated with or without tamoxifen (*n* = 2). **g** Immunoblotting of enteroid lysates from G0 and G4 mice treated with or without tamoxifen with the indicated antibodies (*n* = 2). **h** qRT-PCR analysis of the inflammasome pathway genes from enteroids derived from the G0 and G4 mice treated with or without tamoxifen, pro-IL-18 (*n* = 3). *P* values were calculated using two sided t test between G0 and G4, G4 and G4 + TAM. *P* value between G0 and G4, *p* = 0.0067, between G4 and G4 + TAM, *p* = 0.0063. **i** GSEA plot showing an enrichment of *Yap1* gene signature in the G4 epithelium compared to the G0 epithelium (*p* = 0.006). **j** Overlap of genes identified with YAP1 antibody in G0 and G3 crypt epithelium. **k** Gene enrichment analysis for the differentially bound genes by YAP1 in the G3 epithelium (*n* = 4). **l** ChIP-seq data showing increased YAP1 occupancy at the promoters and the gene body of the inflammasome pathway genes in G3 telomere dysfunctional epithelium compared to the G0 telomere proficient epithelium. **m** Chromatin immunoprecipitation for YAP1 or IgG in the G0 and G3 enteroids treated with or without tamoxifen. qRT-PCR for the indicated genes was performed with primers directed towards the specific site boxed in red and a non-specific site 1Kb away from the YAP1 binding site. (*n* = 3). *P* values were calculated using two sided t test between G0 and G4, G4 and G4 + TAM. *P* value between G0 and G4, *p* = 0.0013, between G4 and G4 + TAM, *p* = 0.0003. *statistically significant, *p* < 0.05 by unpaired Student’s *t* test, two-tailed and Fisher’s Exact Test. *n*, number of mice used in the study. Data are represented as mean ± SEM. Experiments were conducted at least two independent times. Also refer to Supplementary Fig. 2.

Unbiased RNA-seq transcriptomic analyses of isolated colonic epithelial crypts from 3-month old (young) G0, G4 and G4 + TAM mice were conducted to identify gene networks in purified epithelium that may drive inflammation. Gene set enrichment analysis (GSEA) revealed that telomere dysfunction correlated with over-representation of innate immune pathways such as the IFN α and γ pathways and the complement pathway (Fig. 2a, b; *p* = 0.023; enrichment score = 0.62). The IFNγ response pathways were also significantly upregulated in this gene set (Supplementary Fig. 3d). Intersection of (i) differentially expressed genes in young G0 versus G4 crypts, and (ii) differentially expressed genes in young G4 versus G0 and G4 + TAM crypts also highlighted acute reversal of innate immune pathway genes by elimination of telomere dysfunction, with normalization to G0 levels (Supplementary Fig. 3a). Similarly, examination of gene expression changes, specifically in isolated Lgr5 GFP^+^ stem/progenitor cells pooled from the colons of young G0 and G4 mice (Supplementary Fig. 3b), identified inflammatory pathways as top categories (Supplementary Fig. 3c), with the inflammasome pathway as the top differentially regulated pathway (Fig. 2c, d).

Given the prominence of the inflammasome pathway in our model and its known role in intestinal inflammatory disease pathogenesis^16,17^, we evaluated the downstream consequences of the activation of this pathway in the epithelium, namely, caspase-1 cleavage, IL-1β and IL-18 maturation and secretion as read-outs^18–23^. Cytokine array of colonic intestinal epithelial cell lysates as well as quantitation of enteroid RNA levels identified increased IL-1α, IL-1ra, TNF-α and IFN-γ expression in young G4 samples (Supplementary Fig. 3e–g). Since the cytokine array did not include IL-18, ELISA and confirmatory immunohistochemistry were performed, and documented elevated IL-18 levels in G4 colonic lysates and tissue sections; conversely, IL-18 levels were reduced following telomerase reactivation in G4 + TAM colonic lysates and tissue sections (Fig. 2e; Supplementary Fig. 3h). Since caspase-1 cleaves pro-IL-18 to its mature secreted form, we assessed and documented activated caspase-1 (cleaved p20) in the epithelium of telomere dysfunctional mice as well as the mature form of IL-18 and its decreased expression after telomerase reactivation consistent with reduced inflammasome pathway activation (Fig. 2f).

To further confirm intestinal epithelia as the key source of IL-18, which is a known T cell recruitment factor in inflammatory diseases like ulcerative colitis^24–26^, we examined IL-18 levels in pure intestinal epithelial enteroid cultures of young G4 mice (Supplementary Fig. 3i) and confirmed elevated pro-IL-18 protein expression in the epithelial enteroids with telomere dysfunction, and decreased expression upon telomerase reactivation along with extinction of γH2AX signal (Fig. 2g, h; Supplementary Fig. 3j) with no changes in *pro-IL-1β (*Supplementary Fig. 3k*)*. We observed negligible cleavage of pro-IL18 to mature IL18 in vitro (Fig. 2g).

To identify transcription factors responsible for upregulation of pro-IL-18 as well as inflammasome receptors like Nlrp1b, Nlrc4, and Nlrp6, we performed TRANSFAC promoter analysis, and identified several evolutionarily conserved consensus binding motifs, including *NFATc1, HNF4a, NKKX2.3, IRF4/9 and TEF*. The *TEF* binding element emerged as our top priority for further investigation given that its partner, *YAP1*, is a transcriptional co-activator known to be phosphorylated at Y357 by the classical DNA damage signaling molecules ATM and c-ABL^27,28^, leading to YAP1 stabilization and nuclear localization. While acute DNA damage-induced activation of YAP1 can induce cell death^27^, the consequences of YAP1 activation under chronic DNA damage signaling brought about by telomere dysfunction is unexplored, especially in the context of aging and inflammatory diseases. To explore whether YAP1 was driving the phenotype in the telomere dysfunctional mice, we compared the upregulated gene set identified by RNA-seq comparing G0 and G4 epithelium to an established *YAP1* gene signature^29^, revealing activated *YAP1* signature in the G4 epithelium (Normalized Enrichment Score, NES = 1.36 and *p* = 0.006) (Fig. 2i).

To further investigate YAP1 in regulating immune pathways, we performed unbiased ChIP-seq analyses of G0 (telomere intact) and G3 (telomere dysfunctional) colonic crypt tissue. Analysis of the genes bound by YAP1 identified a total of 10,589 genes from the G3 epithelium as compared to 7627 genes from the G0 epithelium. Out of these, only 344 genes overlapped while 7283 and 10245 genes were uniquely bound by YAP1 in the G0 and G3 crypt epithelia, respectively (Fig. 2j). This is consistent with higher YAP1 occupancy of gene promoters in the telomere dysfunctional epithelium, which correlated positively with higher YAP1 expression and stability in the setting of telomere dysfunction. Pathway analysis with GSEA revealed TNF-α signaling via NF-κB to be the topmost pathway in the G3 epithelium, while other pathways related to p53 and apoptosis were also present among the top ten pathways (Fig. 2k). In contrast, several pathways associated with proliferation were among the top 10 in G0 (Supplementary Fig. 3l). Notably, we confirmed higher YAP1 occupancy at the *NLRP1B, NLRP6, NLRC4*, as well as *IL-18* gene promoters in G3 versus G0 epithelium (Fig. 2l). Finally, anti-YAP1 ChIP validated YAP1 binding to the promoter region of pro-IL-18 with elevated binding observed in G3 enteroids relative to G0 controls and, conversely, reduced binding in G3 + TAM samples (Fig. 2m). Together, these unbiased analyses support a prominent role for YAP1 in driving inflammation in the context of telomere dysfunction.

### Telomere dysfunction activates YAP1

**Fig. 3:**
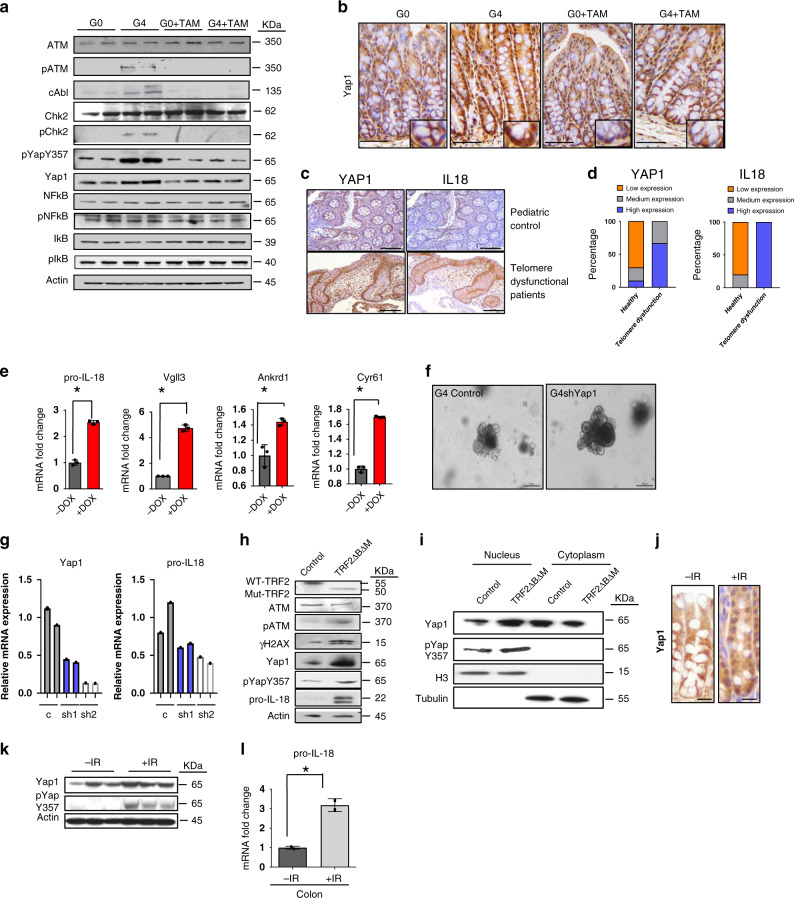
Telomere dysfunction activates YAP1. **a** Immunoblotting of intestinal enteroid lysate from G0 and G4 mice treated with or without tamoxifen for the indicated antibodies (*n* = 2). **b** Immunohistochemistry for YAP1 in the colonic crypts from the G0 and G4 mice treated with or without tamoxifen (*n* = 3). Scale bars, 50 µm. Insets are shown to illustrate the higher nuclear localization of YAP1 in the G4 mice than in others. **c** Immunohistochemistry with the colonic epithelium from healthy pediatric patients(control) and patients with telomeropathies for YAP1 and IL-18. (Control, *n* = 10; telomere dysfunctional patients, *n* = 3) **d** Histogram depicting the quantification of staining intensity for YAP1 and IL-18 in the colonic epithelium of healthy (control) and telomere dysfunctional patients. YAP1, *p* = 0.0035 IL-18, *p* = 0.0001. (Control, *n* = 10; telomere dysfunctional patients, *n* = 3). **e** qRT-PCR for the indicated genes from enteroids isolated from the YAP1(S127A) transgenic mouse treated with or without doxycycline to inhibit degradation and increase stabilization and nuclear localization (*n* = 3). *P* values were calculated using two sided t test between dox untreated and dox treated organoids. The following are the *p*-values for pro-Il18, *p* < .0001, Vgll3, *p* = 0.0067, Cyr61, *p* < 0.0001. **f** Microscopic images of the enteroids from a control vector transduced or *YAP1* shRNAs transduced enteroids (*n* = 2). Scale bars, 30 µm. **g** qRT-PCR for the indicated genes from G4 enteroids transduced with either a control or *YAP1* shRNAs (*n* = 2). Histogram shown with individual bar for each sample. **h** Western blot for the indicated proteins in CRL-1831 cells overexpressing TRF2ΔBΔM plasmid or control vector 1-day post selection with puromycin. **i** Cell fractionation performed with CRL-1831 cells overexpressing TRF2ΔBΔM plasmid or control vector 1-day post selection with puromycin. **j** Immunohistochemistry for YAP1 in irradiated or control G0 colon crypts showing higher nuclear positive staining for YAP1 after irradiation. Scale bars, 50 μm. **k** Western blot for the YAP1 and pYAPY357 phosphorylated proteins in irradiated or control G0 colon crypts showing higher levels of phosphorylation after irradiation (*n* = 3). **l** qRT-PCR of RNA from control or irradiated colonic G0 mouse crypts for pro-IL-18 (*n* = 3). *P* values were calculated using two sided *t* test, *p* = 0.0122. *statistically significant, *p* < 0.05 by unpaired Student’s *t* test, two-tailed and Fisher’s Exact Test. *n* represents number of mice used in the study. “Ab” denotes antibody and “I” denotes Input samples. Each experiment was conducted at least two times. Data are represented as mean ± SEM. Also refer to Supplementary Fig. 3.

In our model system, higher levels of phosphorylated ATM, CHK2, and YAP1(Y357) were detected in telomere dysfunctional enteroids, and these signals were reduced upon telomerase reactivation (Fig. 3a). While NF-κB serves as a master transcriptional regulator for many of the inflammatory genes, we documented no changes in the phosphorylation of key NF-κB pathway components (pNF-κB, IκB and pIκB) across these samples (Fig. 3a). In G4 intestines, higher levels of YAP1 nuclear localization were detected in the colonic crypts (both stem and progenitor cells) (Fig. 3b) similar to the IL-18 secretion pattern (Supplementary Fig. 3g); and YAP1 nuclear signal diminished upon telomerase reactivation (Fig. 3b). Importantly, patient biopsies from Texas Children’s Telomere Registry revealed significant stabilization of YAP1 and upregulation of IL-18 levels in the colonic epithelium (Fig. 3c, d; Supplementary Fig. 4a), further supporting the model that telomere dysfunction activates this pathway to drive inflammation.

The role of YAP1 was genetically validated with enteroids derived from doxycycline-inducible R26-Yap1 transgenic mice^30^ showing that elevated transgene expression of *Yap1* (Supplementary Fig. 4b) drove higher expression of pro-IL-18 as well as Vgll3, Ankrd1 and Cyr61 (direct YAP1 targets) compared to un-induced enteroids from the same transgenic strain (Fig. 3e). Conversely, shRNA mediated YAP1 depletion in telomere dysfunctional enteroids showed reduced expression of *pro-IL-18* compared to vector control (Fig. 3f, g; Supplementary Fig. 4d–f; Supplementary Table 4).

Inflammasome activation is a key cooperating factor that induces cleavage of procaspase-1 to caspase-1, which in turn cleaves pro-IL-18 to mature IL-18^31^. Further, *Yap* ChIP-seq data from our study as well as the Gregorieff et al. study^29^ also demonstrated that *IL-18*, *Nlrc4, Nlrp1b* and *Nlrp6* along with several other inflammasome genes were YAP1 targets, many of which were also differentially expressed in our RNA-seq dataset. We therefore examined the expression of key microbial receptors capable of assembling inflammasomes. In our model, we observed increased expression of key inflammasome proteins (Nlrp1b, Nlrp3, Nlrc4 and Nlrp6) in the telomere dysfunctional enteroids and their decline upon telomerase reactivation (Supplementary Fig. 4g–j). In addition, reduced expression of microbial receptors in the *YAP1* shRNA treated G4 enteroids (Supplementary Fig. 4f) and increased expression of microbial receptors and inflammasome pathway genes *Nlrp1b*, *Nlrp3*, *Nlrc4* and *Nlrp6* were also observed in the YAP1-activated enteroids (Supplementary Fig. 4c).

To obtain additional genetic evidence for telomere dysfunction-driven YAP1 activation, enforced expression of dominant negative TRF2(ΔBΔM) in the spontaneously immortalized colon cell line CRL1831 was accompanied by increased pγH2AX, pATM, pYAP1(Y357) and pro-IL-18 levels (Fig. 3h). Cell fractionation studies further documented increased pYAP1(Y357) nuclear localization (Fig. 3i). Moreover, moderate ionizing radiation (IR) (2 Gy/day × 5 days) showed elevated YAP1 nuclear localization in the colonic crypt epithelium consistent with previous reports (Fig. 3j) and increased phosphorylation of YAP1 at Y357 (Fig. 3k). In these IR-exposed crypts, we documented elevated *pro-IL-18* mRNA levels (Fig. 3l) and increased levels of *Nlrp1b, Nlrp3, Nlrc4, and Nlrp6* mRNA (Supplementary Fig. 4k–n). We conclude that DNA damage signaling from telomeres can stabilize and activate YAP1 which in turn directly elevates transcription of key inflammasome genes and pro-IL-18.

### Inhibition of ATM and YAP1 reduces IL-18 secretion and ameliorates inflammation

**Fig. 4:**
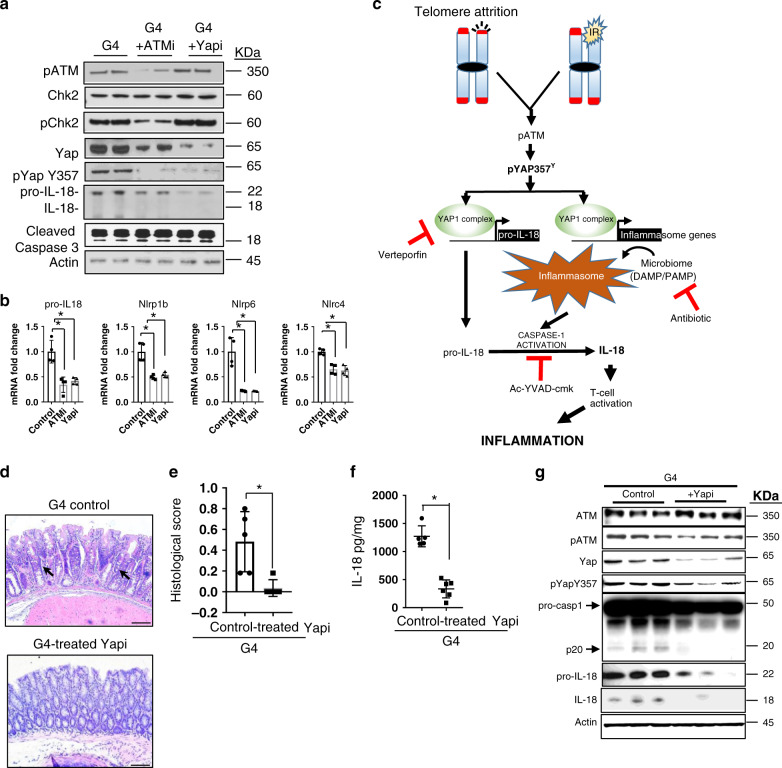
Inhibition of ATM and YAP1 reduces IL-18 secretion and ameliorates inflammation. **a** Immunoblots of intestinal enteroid lysate from G4 enteroids treated with or without ATM or YAP1 inhibitors for the indicated antibodies (*n* = 2). **b** qRT-PCR for the indicated genes with RNA from enteroids treated with DMSO only or inhibitors for ATM or YAP1 (*n* = 4). *P* values were calculated using two sided *t* test. The following are the *p*-values, pro-IL18, *p* = 0.0030, Nlrp1b, *p* = 0.0018, Nlrp6, *p* = 0.0015, Nlrc4, *p* = 0.0010. **c** Model representing the telomere dysfunction-mediated inflammasome pathway and the nodes at which the inhibitors for YAP1, caspase-1 or antibiotics inhibit the pathway leading to reduced secretion cleavage of caspase-1 and secretion of IL-18 by the colonic epithelium. **d** Hematoxylin and eosin staining of colon tissue from G4 mice treated with or without the YAP inhibitor, verteporfin. (control, *n* = 5; inhibitor treated, *n* = 6). Scale bars, 50 μm. **e** Histopathological score of the intestinal phenotype of the G4 mice treated with or without verteporfin (Yapi) (control, *n* = 5; inhibitor treated, *n* = 6). *P* values were calculated using two sided t test, *p* = 0.0048. **f** ELISA for the quantification of mature IL-18 from the colonic epithelium of the G4 mice treated with or without verteporfin (Yapi) (control, *n* = 5; inhibitor treated, *n* = 6).*P* values were calculated using two sided *t* test, *p* < 0.0001. **g** Western blot of colonic lysate from the colonic epithelium of the G4 mice treated with or without verteporfin (Yapi), for the indicated antibodies (*n* = 3). *statistically significant, *p* < 0.05 by unpaired Student’s *t* test, two-tailed. *n* represents number of mice used in the study. Data are represented as mean ± SEM. Experiments were conducted at least two independent times.

To validate the functional and translational relevance of ATM-YAP1 signaling in the regulation of pro-IL-18 expression, we treated G4 enteroids with ATM (0.1 μM × 24 h) or YAP1 inhibitors (4 μM × 24 h) and assessed pro-IL-18 levels. While this brief treatment did not impair cell viability (as determined by cleaved caspase-3), significant reductions were observed in pYAP1(Y357) levels and pro-IL-18 levels, demonstrating that ATM and YAP1 are active in the G4 enteroids with telomere dysfunction and can drive transcription of pro-IL-18 (Fig. 4a, b).

Since enhanced secretion of IL-18 in the colonic epithelia is thought to be damaging and possibly an inducer of inflammation, we considered the possibility that reduction of IL-18 via inhibition of its upstream inducer could serve as a potential therapeutic strategy (Fig. 4c). Thus, in addition to the above genetic validation, we pharmacologically inhibited YAP1 with verteporfin or caspase-1 inhibitor Ac-YVAD-cmk^32^ in three-month old G4 mice, resulting in reduced inflammation when compared to age-matched controls (Fig. 4d, e; Supplementary Fig. 5c, d), reduced epithelial IL-18 expression (Fig. 4f, g; Supplementary Fig. 5e, f) and lower IFN-γ expression (Supplementary Fig. 5a, g). Reduction in YAP1 and pYAP1(Y357) levels were detected in the treated mice along with a reduction in pro-IL-18 and mature IL-18 levels (Fig. 4g; Supplementary Fig. 5f). Notably, decreased procaspase-1 cleavage was also observed in the verteporfin treated cohort, which reinforced that YAP1 contributes to inflammasome activation through transcriptional regulation of key microbial receptors involved in inflammasome assembly and procaspase-1 cleavage (Fig. 4g). Accordingly, verteporfin treatment resulted in reduced expression of several inflammasome pathway genes involved in inflammasome assembly (Supplementary Fig. 5b).

The intestinal microbiome has been shown to play a major role in the induction of intestinal inflammation by virtue of its interaction with both the epithelial and the immune compartments and induction of caspase-1 cleavage. Furthermore, the greater disease severity in the G4 colon relative to the small intestine is consistent with diminished microbial load in the small intestine relative to the colon (Supplementary Fig. 6a)^33,34^ and minor differences in the microbiome composition in the G0 and G4 mice (Supplementary Fig. 6b). To explore the gut microbiome as a potential cooperating factor with telomere dysfunction in the induction of IBD, we treated the mice with broad spectrum antibiotics (trimethoprim-sulfamethoxazole) in drinking water to reduce the gut microbial load (Supplementary Fig. 6c). G4 mice exhibiting epithelial ulcerations as assessed by colonoscopy were treated with antibiotics for one month. Compared to the untreated controls, the treated cohorts exhibited significant reductions in disease pathology (Supplementary Fig. 6d, e), pro-IL-18 and mature IL-18 levels (Supplementary Fig. 6f, g), caspase-1 cleavage (Supplementary Fig. 6g) and IFN-γ secretion (Supplementary Fig. 6h). Together, these data support a model in which telomere dysfunction-mediated YAP1 phosphorylation and transcriptional activation, and gut microbiome-mediated caspase-1 cleavage converge on the elevated production of mature IL-18 leading to the recruitment of IFN-γ secreting T cells to initiate and maintain inflammation.

## Discussion

In this study, human and murine analyses identified a mechanistic basis of the enigmatic link between defective telomere maintenance and inflammatory disease. We show that telomere dysfunction activates ATM/YAP1 to up-regulate pro-IL-18 in epithelial cells. This pathway cooperates with caspase-1 leading to the elevated secretion of mature IL-18 to induce IFN-γ secretion by resident T cells to perpetuate considerable tissue inflammation and damage. Pharmacological inhibition of ATM in vitro, pharmacological and genetic inhibition of YAP1 in vitro and pharmacological inhibition of YAP1 or caspase-1 in vivo suppressed IL-18 secretion, mitigating inflammation. Given that the telomere dysfunctional mice display intestinal inflammatory phenotype that can be ameliorated by inhibiting YAP1 or caspase-1, these strategies may also inform potential therapeutic opportunities for other inflammatory diseases.

Findings presented here challenge current dogma that telomere damage is a mere consequence of inflammation and instead assign telomere dysfunction as a prime pathogenic driver of the inflammatory process. Along these lines, our own data on patients with germline mutations in telomere-related proteins as well as a recent study of the gastrointestinal manifestations in younger patients with mutations in *TERT*, *TIN2, TRF2,* or *TR* genes revealed significant pancolitis, villous atrophy, crypt dropout and intraepithelial lymphocytosis as the most common lower GI tract manifestations^7^. Finally, several studies have positively correlated short telomeres in the intestinal epithelium of ulcerative colitis patients to disease severity, raising the possiblity of a feedforward loop accelerating telomere dyfunction and increasing inflammation^10,35^. Thus, our mechanistic and murine genetic studies, together with these correlative clinical observations, strongly implicate telomere dysfunction as a key pathogenetic factor in chronic intestinal inflammatory disorders. In the *Lgr5-CreERT*^2^ model, this allele exhibits mosaic expression and hence regional rescue of the intestinal degenerative phenotype. This partial restoration of intestinal compromise, coupled with persistent degenerative phenotypes across all other organ systems, likely accounts for the modest increase in survival compared with our previous study of widespread telomerase reactivation^13^. Also, while we observe robust reversal of intestinal inflammation with intestine epithelial-specific telomerase reactivation, it is also possible that a broader systemic activation of telomerase would have an even greater anti-inflammatory therapeutic benefit given the persistent presence of the senescent stroma in the intestine. The robustness of our rescue, coupled with the fact that senescent stroma is known to release pro-inflammatory cytokines^36,37^, emphasizes the centrality of the intestinal epithelium in our model. Future studies should consider the use of Cre-expressing models that exhibit universal activity in all intestinal epithelial cells for more complete epithelial telomerase reactivation (e.g., *LSL-mTert x Olfm4-IRES-eGFPCreERT2* or *Villin-CreERT2*) as well as crosses that assess telomerase reactivation in the stroma (e.g., *LSL-mTERT x αSMA-CreERT2*).

A significant finding of our work is the key role of YAP1 in the control of *pro-IL-18* gene expression. The function of YAP1 as a driver of intestinal inflammation is intriguing given the presence of a SNP in the *TEF* locus (TEF is a transcriptional co-factor of Yap1) in IBD patients^38^. This is also reinforced by the recent work from the Karin laboratory identifying YAP overexpression in IBD patient biopsies^39^. Thus, we speculate that our model could provide a system to test preventive and therapeutic strategies for this obdurate medical condition, and identifies YAP or IL-18 inhibition as viable therapeutic targets of intestinal inflammation. In conclusion, our comparative murine and human analyses reveal a direct role for telomere dysfunction in inflammation, shedding light on the basis of increased incidence of inflammatory diseases in individuals with germline mutations in telomere maintenance genes.

## Methods

### Human subjects

The investigation of patients with a telomere biology disorder was conducted according to Declaration of Helsinki Principles. Informed consent was received from participants prior to inclusion in the study according to protocol H-17698 Genetic and Biological Determinants of Bone Marrow Failure approved by the Institutional Review Board for Baylor College of Medicine and affiliated hospitals. The subjects investigated for the purpose of this study included the subset (*n* = 21) with a known diagnosis of a telomere biology disorder.

### Mice

The *LSL-mTert* allele and the *Lgr5-EGFP-IRES-CreERT*^2 ^^13,40^ have been described elsewhere. The *TetO-YAP(S127A)*^30^ used in the paper was generated by Fernando Camargo at Harvard University. The mice were housed in a barrier facility, at housing temperature of 25 °C under ambient oxygen conditions in a 12 h light/12 h dark cycle under 50% humidity.

### Animal Experiment

All mouse manipulations were reviewed and approved by MD Anderson Cancer Center’s Institutional Animal Care and Use Committee (IACUC). All animals were maintained in pathogen-free conditions and cared for in accordance with policies and certification of the Association for Assessment and Accreditation of Laboratory Animal Care International (AAALAC International). Two-month old or six-month old telomere dysfunctional mice were treated with 100 ul of 10 mg/ml tamoxifen (Sigma) dissolved in corn oil (Sigma) by intraperitoneal injection daily for 5 days to activate telomerase. For the antibiotic treatment, colonoscopy was performed on telomere dysfunctional mice at 4–5 months of age to monitor inflammation. For the YAP1 inhibitor treatment, telomere deficient G4 mice were treated intraperitoneally with either the YAP1 inhibitor verteporfin (Selleckchem, 100 mg/kg in PBS) or vehicle for 14 alternate days. At the end of 14 days, both verteporfin treated and untreated mice were euthanized, and intestines were collected and immediately fixed in formalin. The G4 telomere dysfunctional mice were treated with the caspase-1 inhibitor Ac-YVAD-cmk at 1.25 mg/kg for 2 weeks. At the end of two weeks the mice were euthanized, and intestines were collected. The G4 mice with signs of colonic inflammation were treated with or without TMS (Trimethoprim Sulfa, Sigma, 0.13 mg/ml trimethoprim and 0.67 mg/ml sulfa) in drinking water for one month. At the end of one month the mice were euthanized and their intestines collected.

### Histologic analysis and Immunohistochemistry and immunofluorescence

Mouse intestines were prepared as swiss rolls to facilitate histological assessment of the full length of the intestine. Intestinal tissues were fixed in 10% neutral buffered formalin, paraffin embedded, cut into 5 µm sections and stained with hematoxylin and eosin. A blinded histology assessment was performed by two independent pathologists scoring them from 0 to 5 was assigned based on the following criteria: cryptitis, crypt abscess, erosion, ulcer, goblet cell depletion, increased chronic inflammation in the lamina propria, basal plasmacytosis, submucosal inflammation, Paneth cell metaplasia and crypt distortion. An average was calculated based on the scoring for each animal. These averages were plotted in GraphPad Prism v8 and histograms generated. Immunohistochemistry was performed by an established protocol^41^. Specifically, the slides were baked at 60 °C for 1 h and passed through a gradient of xylenes and ethanols for deparaffinization and rehydration. Antigen unmasking was done in citrate buffer pH6.0 in a poressure cooker. Non-specific signals were blocked using 3% BSA, 10% goat serum in 0.1% Triton X-100. Intestinal tissue samples were stained using the following antibodies: GFP (D5.1, Cell Signaling Technologies, 1:200, #2956, Abcam, 1:500, ab13970), γH2AX (Ser139) (Millipore, 1:500, 05-636), YAP (Novus Biologicals, 1:200, NB110-58358), IL-18 (Abcam, 1:200, ab71495) and (Sigma 1:200, HPA003980), IFN-γ (Abcam,1:1000, ab183685), caspase-1 (Abcam, 1:200, ab108362).

### Histopathology assessment of the patient biopsies

Three biopsies from pediatric patients with telomereopathies were obtained from Dr. Alison Bertuch, from the Telomere Registry at Texas Children’s. For immunohistochemistry, the epithelial cells at each intensity of staining were recorded on a scale of 1 (weak staining = low expression), 2 (moderate staining = medium expression), and 3 (strong staining = high expression). Biopsies from each patient were assessed after examining three different microscopic field for each biopsy and scores were assigned by two independent board-certified pathologists in a blinded manner.

### γH2AX quantification

Positive signal for γH2AX(Ser139) was counted in each whole crypt both in the stem cells and the progenitor population of the intestine and was determined from an average of 100 crypts from three different intestinal sections from three mice.

### GFP positive crypt quantification

One hundred crypts were audited for the presence of GFP positive cells in the crypts from five different mice 8-month old for G0 and G4 treated with or without tamoxifen. Percentage was calculated. Two hundred (200) GFP positive crypts were also audited for the presence of γH2AX expression from five different mice 8-month old for G0 and G4, treated with or without tamoxifen.

### Flow cytometry

Colonic lamina propria lymphocytes were isolated from telomere deficient or proficient mice treated with or without tamoxifen according to a well-established protocol^42^. Specifically, the intestinal tissue was removed of fat and mesentery, then washed briefly and incubated in 2 mM EDTA in PBS for 30 min at 37 °C to get rid of the crypt epithelium. The tissue was chopped into pieces and digested by incubation in 0.2 mg/ml collagenase Type VIII (Sigma) and DNAse I (Sigma) for 10 min at 37 °C with rocking. The cells were filtered through 100 µm and stained with the following BioLegend antibodies: CD45 (APC-Cy7), CD3 (BV510), CD4 (PECy7), CD8 (AF700), CD11b (BV605), B220 (PE), F4/80 (PerCP) for 30 min on ice in the dark. Data were analyzed using FlowJo v10.

### RNA isolation and qRT-PCR

Colonic epithelial cells were isolated by incubating the tissue with 2 mM EDTA at 4 °C for 30 min. RNA was isolated by directly adding RLT buffer of the RNeasy Mini Kit (Qiagen) to wells containing 100 enteroids. DNA was removed by treating with DNase from the kit (Qiagen). RNAqueous-Micro Total RNA Isolation kit (Invitrogen) was used to isolate RNA from the GFP-sorted stem/progenitor cells. cDNA was generated using the Invitrogen superscript kit. qRT-PCR was performed with 1x SYBR Green PCR Master Mix (Applied Biosystems), using primers that had been optimized for their melt curve (see Supplementary Table 3). Glyceraldehyde 3-phosphate dehydrogenase (GAPDH) expression was used as a positive control.

### RNA-seq

RNA was isolated as described above. RNA-seq was performed by the Sequencing and Microarray Facility (SMF) core at MD Anderson. Libraries were generated using Illumina’s TruSeq kit and were sequenced using the Illumina HiSeq2000 Sequencer. Raw sequencing data (BCL format) were converted to Fastq files using Illumina Casava software (v1.8.2) and aligned to the mouse reference genome (mm10) using STAR software. The HTSeq-count program was used to generate raw read counts for each gene. The R package edgeR was used for data normalization and differential expression analysis using the criteria of log2 fold change ≥ 0.5 or ≤−0.5 and p value ≤ 0.1. Pathway enrichment analysis was performed using GSEA software based on p value from the aforementioned differential expression analysis, and clustering visualization was done by the R package heat map. To analyze YAP signature genes, the list of YAP signature genes 29 was obtained and formatted as the compatible pathway database (grp format) with GSEA. RNA from mouse colonic epithelial cells were isolated and sequenced using the Illumina HiSeq2000 Sequencer. In addition, we obtained a 92 IBD gene signature panel (common between the colons of ulcerative colitis and Crohn’s disease patients).

### Enteroid culture and treatment

Intestinal enteroids were cultured according to an established protocol by Sato and Clevers^43^. Specifically, the ileum was opened longitudinally, and crypts isolated by incubating in cold PBS with 2 mM EDTA for 30 min and brief shaking by hand. The crypts were then filtered through a 70 µm filter and embedded in Matrigel. The Matrigel was layered with Advanced DMEM/F12 media supplemented with 2 mM GlutaMax (Invitrogen), 100 U/ml Penicillin, 100 mg/ml Streptomycin (Invitrogen), N2 Supplement (Invitrogen), B-27 Supplement (Invitrogen), mouse recombinant EGF (R&D Systems), 100 ng/ml mouse recombinant Noggin (Peprotech), 500 ng/ml human RSP1 (R&D Systems). Enteroids were cultured for 5 days with or without tamoxifen (0.2 µg/ml); telomerase induction and γH2AX levels were validated. G0 and G4 enteroids were always compared from the same passage and experiments were all conducted with enteroids earlier than passage 10. Equal number of G0 and G4 enteroids from the same passage treated with or without tamoxifen were plated in 30 µl of Matrigel in each well of a 24-well plate. For lentiviral shRNA transduction, the enteroids were cultured in high WNT-containing media for 48 h with CHIR99021 (1 µm, STEMCELL Technologies) and Nicotinamide (10 µM, Sigma). Viral pellet was harvested by ultracentrifugation and dissolved in media containing Jagged-1 peptide (1 µM, Anaspec), Nicotinamide (10 µM, Sigma), N-acetylcysteine (1 µm, Sigma), CHIR99021 (1 µM, STEMCELL Technologies), Y-27632 (1 µM, STEMCELL Technologies) and polybrene (1:1000, Millipore). The enteroids were digested with TrypLE (Gibco) and spinoculation was done with the virus at 600 × *g* for 45 min at 32 °C in a 48-well plate. The enteroid -viral mixture was incubated at 37 °C for 3 h and plated in Matrigel dome covered with the spinoculation media for 2 days. Afterwards the enteroids were selected with 2 µg/ml puromycin for the entire duration of the experiment. Enteroids were further treated with inhibitors for ATM (KU-60019, 0.1 μM for 24 h) and YAP1 (verteporfin, 4 µM for 24 h) For Western blotting, enteroids from the same passage were harvested at day 2/3 and Matrigel was dissolved in ice-cold media. Protein was collected by digesting in RIPA buffer with protease cocktail and phosphatase inhibitors.

### Western blotting and antibodies

Enteroids were washed with cold PBS to remove Matrigel. The pellets were then lysed in RIPA buffer containing proteinase and phosphatase inhibitors and incubated on ice for 30 min. Lysates were centrifuged at 13,000 × *g* for 15 min at 4 °C. Protein concentrations were measured using the DC protein assay kit (Bio-Rad). SDS-PAGE and immunoblotting were carried out in pre-cast bis-Tris 4–20% gradient gels and 3–8% Tris-acetate gradient gels (Invitrogen). The following antibodies were used: anti-phospho-histone H2AX (Ser139) (Millipore, clone JBW301, 05-636, 1:1000), ATM (Novus Biologicals, NB100-104, 1:500), pATM (Ser1981; Novus Biologicals, NB100-306, 1:500), c-ABL (Cell Signaling, #2862, 1:1000), CHK2 (Cell Signaling Technologies, catalog #2662,1:1000), pCHK2(Thr68), (Cell Signaling technologies, 2661,1:1000), total YAP1 (Novus Biologicals, NB110-58358, 1:1000), pYAP1(Y357) (Abcam, ab62751, 1:1000), NF-kB (Cell Signaling, #9936, 1:1000), pNF-kB (Cell Signaling, #9936, 1:1000), IkB (Cell Signaling, #9242, 1:1000), pIkB (Cell Signaling, #9246,1:1000), IL-18 (Abcam, 1:1000, ab71495), pro-IL-18 (Proteintech, 10663-1-AP, 1:1000), mature IL-18 (MBL International corporation, clone 39-3F, 1:1000), cleaved caspase 3 (Cell Signaling, #9661, 1:1000), caspase-1 (p20) (Adipogen, AG-20B-0042-C100H3, 1:1000), H3 (Sigma, 1:1000, H0164), H2A.X (Proteintech, 1:1000, 10856-1-AP), tubulin (Sigma, 1:1000, T9026) and actin (Sigma, 1:5000). The raw uncropped images for western blot data are available in the source data file.

### Cells and plasmids

CRL1831 cell line was purchased from ATCC and cultured according to the manufacturer’s instructions. TRF2ΔBΔM cloned into the pLPC vector and the vector control plasmids were purchased from Addgene (Plasmid #18008, Plasmid #12540). Retroviruses were packaged in 293T cells using pVSV-G and pHIT60. All the lentiviral shRNA plasmids used were cloned into the pLKO vector and the vector controls were obtained from Sigma Aldrich. Lentiviruses were packaged in 293T cells using second generation packaging vectors, psPAX2 (Addgene plasmid 12260) and pMD2.G (Addgene plasmid 12259). The lentiviral shRNAs used in this study are listed in Supplementary Table 4.

### Cell fractionation

Cell fractionation was performed according to the manufacturer’s protocol (Thermo Scientific, Subcellular protein fractionation kit for cultured cells). Western blot was performed with equal amounts of protein loaded from each fraction.

### Chromatin immunoprecipitation and ChIP-seq assay

ChIP was performed following an established protocol^41^. qRT-PCR was performed with the following primer sequences specific for pro-IL-18, murine-3880, 5′ TTCAAAGCGGGTCTTTGGGA 3′ (forward) and 5′ CACTGATAGAGCCTGTGGGC 3′ (reverse) and non-specific, −879, 5′ GTAATCCAGCACTGGGTGGT 3′ (forward) and 5′ TTGGCTAGCTAAGCGGATGAG 3′ (reverse). ChIP-seq assays were performed^44^. Specifically, ~2 × 10^7^ large intestinal crypt cells were harvested via cross-linking with 1% (wt/vol) formaldehyde for 10 min at 37 °C with shaking. After quenching with 150 mM glycine for 10 min at 37 °C with shaking, cells were washed twice with ice-cold PBS and frozen at −80 °C for further processing. Cross-linked pellets were thawed and lysed on ice for 30 min in ChIP harvest buffer (12 mM Tris-Cl, 1 × PBS, 6 mM EDTA, 0.5% SDS) with protease inhibitors (Sigma). Lysed cells were sonicated with a Bioruptor (Diagenode) to obtain chromatin fragments (~200–500 bp) and centrifuged at 15,000 × *g* for 15 min to obtain a soluble chromatin fraction. In parallel with cellular lysis and sonication, antibodies (5 μg/ 3 × 10^6^ cells) were coupled with 30 μl of magnetic protein G beads in binding/blocking buffer (PBS + 0.1% Tween + 0.2% BSA) for 2 h at 4 °C with rotation. Soluble chromatin was diluted five times using ChIP dilution buffer (10 mM Tris-Cl, 140 mM NaCl, 0.1% dissolved organic compound, 1% Triton X, 1 mM EDTA) with protease inhibitors and added to the antibody-coupled beads with rotation at 4 °C overnight. After washing, samples were treated with elution buffer (10 mM Tris-Cl, pH 8.0, 5 mM EDTA, 300 mM NaCl, 0.5% SDS), RNase A, and Proteinase K, and cross-links were reversed overnight. ChIP DNA was purified using AMPure XP beads (Agencourt) and quantified using the Qubit 2000 (Invitrogen) and Bioanalyzer 1000 (Agilent). Libraries for Illumina sequencing were generated following the New England BioLabs (NEB) Next Ultra DNA Library Prep Kit protocol. A total of 10 cycles were used during PCR amplification for the generation of all ChIP-seq libraries. Amplified ChIP DNA was purified using double-sided AMPure XP to retain fragments (~200–500 bp) and quantified using the Qubit 2000 and Bioanalyzer 1000 before multiplexing.

### ChIP-seq data processing

Raw fastq reads for all ChIP-seq experiments were processed using FastQC (http://www.bioinformatics.babraham.ac.uk/projects/fastqc/), and quality reads were aligned to the mm9 reference genome using Bowtie version 1.2.2^45^ with the following criteria: --best --chunkmbs 320. To directly compare G0 and G3 ChIP-seq samples, uniquely mapped reads for each mark were normalized by total reads per condition, sorted, and indexed using Samtools version 1.9^46^. Model-based analysis of ChIP-seq (MACS) (version 1.4.2)^47^ was used to identify YAP1 enrichment over “input” background. MACS2 was used to identify the differential binding of YAP1 in G0 and G3 with the following criteria: bdgdiff –g 60 –l 120. To visualize ChIP-seq libraries on the IGV browser, we used deepTools version 2.7.15 to generate bigWig files by scaling the bam files to reads per kilobase per million (RPKM) using the following criteria: bamCoverage –b–normalizeUsing RPKM–smoothLength 300–binSize 30–extendReads 200 –o.

### Microbiome analysis

Microbiome community studies were performed at the Alkek Center for Metagenomics and Microbiome Research at Baylor College of Medicine, Houston, Texas. DNA was extracted from fecal pellets, and the 16S rRNA V4 region was amplified by PCR. Products were sequenced using an Illumina MiSeq platform and a 2 ×250 bp paired-end protocol. 16S rRNA gene sequences were assigned into operational taxonomic units (OTUs) using the UPARSE pipeline and alignment to the SILVA SSURef_NR99_119 database at 97% sequence identity. Analysis and visualization of microbiome communities were conducted with the publicly available software R (R Core Team 2015, version 3.2.2), utilizing the phyloseq package (Bioconductor) to import sample data, calculate α- and β-diversity metrics, and microbiome community profiles. For the 16S rRNA gene quantification, bacterial DNA was extracted by a previously described method using the MO BIO PowerSoil DNA Isolation Kit (MO BIO Laboratories) following the manufacturer’s instructions and quantified using Qubit (Life Technologies). Quantitative PCR was performed in QuantStudio 7 Real-Time PCR System using PerfeCTa SYBR Green Fast Mix. The qPCR primers (1369F-1492R) target regions flanking V9 of the 16S rRNA gene. A standard curve was made using a serially diluted plasmid that contained nt 1369 to 1492 of an *E. coli* 16S rRNA gene. The concentrations of unknowns were calculated from C_T_ values using the equation generated from plotting the standard curve. All samples were run in triplicate, including the standard curve, a set of non-template controls (NTC), and inhibitor controls (known positives + unknown DNA).

### ELISA with tissue lysate and cytokine array

ELISAs for IL-18 were performed with mouse colonic epithelial tissue lysate using the ELISA kit (MBL International Corporation) following manufacturer’s instructions. For the ELISA from the inhibitor treated animals, half of the colon containing the distal intestine was fixed and stained for H&E for further pathological analysis, epithelial cells were isolated by incubation in 10 mM EDTA at 4 °C to obtain epithelial cells which were subsequently lysed for Western blot. The remaining lamina propria was enzymatically digested and immune cells isolated by previously described method for flow cytometric analysis42. Cytokine array was performed with colonic epithelial cell lysates from G0 and G4 mice at 3 months of age from different animals. Equal amounts of lysate were loaded onto the array according to the manufacturer’s protocol, incubated with antibody mix overnight and developed the next day. The spots were quantified with ImageJ software and the top four upregulated and three downregulated cytokines are denoted.

### Statistical analysis

GraphPad Prism 8 software was used to conduct the statistical analysis of all the data. Data are represented as mean and SEM. All experiments were done in triplicate unless otherwise specified. Student’s unpaired *t* test, two tailed was used for comparison between two groups, and *p* ≤ 0.05 was considered significant. Survival curve analysis was determined by Kaplan–Meier analysis. Fisher’s Exact Test for Count data were used to calculate significance for the patient biopsy immunohistochemistry.

### Reporting summary

Further information on research design is available in the Nature Research Reporting Summary linked to this article.

## Supplementary information

Supplementary Information

Reporting Summary

## Data Availability

The RNA-seq data are available at “GSE108902”. The ChIP-seq data are available at “GSE144200”. Source data are provided with this paper. Any other data or information are available from the corresponding author on request. Source data are provided with this paper.

## Source data

Source Data
