# Supplementary material for: Telomere dysfunction activates YAP1 to drive tissue inflammation
Source: Nat Commun. 2020 Sep 21;11:4766. doi: 10.1038/s41467-020-18420-w (PMC7505960; doi:10.1038/s41467-020-18420-w)
Supplement: Supplementary file 1 — Supplementary Information [file 41467_2020_18420_MOESM1_ESM.pdf]

Supplementary Information

Telomere dysfunction activates YAP1 to drive tissue inflammation

Chakravarti et.al.

Supplementary Figure 1

Telomere dysfunction in the gut epithelia drives inflammation

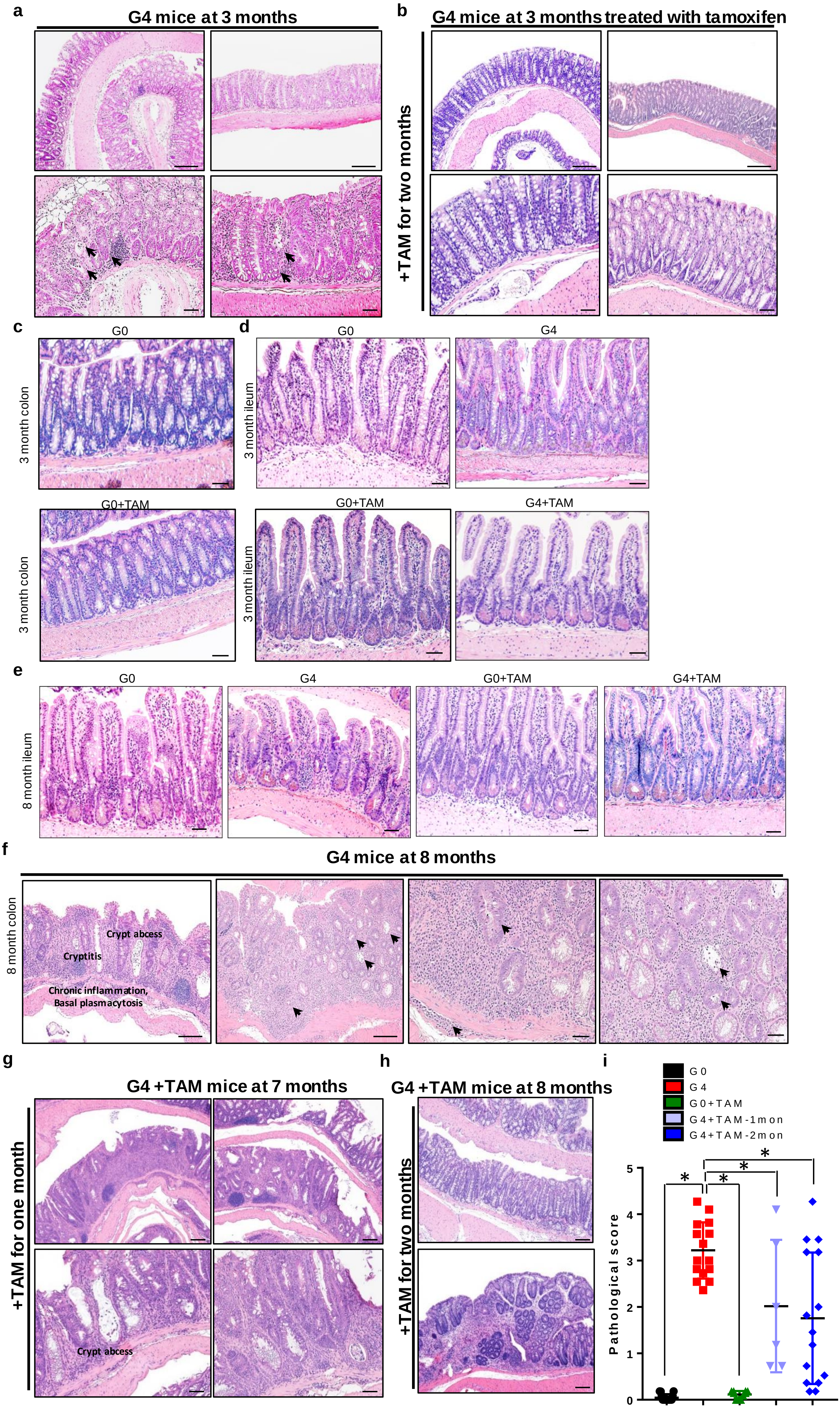

Supplementary Figure 1. Telomere dysfunction in the gut epithelia drives inflammation

a. Hematoxylin and eosin staining of colon tissue from 3-month old G4 mice (n = 15). Scale bars, 100µm. Arrows indicate cryptitis and crypt abscess.

b. Hematoxylin and eosin staining of colon tissue from 3-month old G4 mice treated with tamoxifen (n = 15). Scale bars, 100µm.

c. Hematoxylin and eosin staining of colon tissue from 3-month old G0 mice treated with or without tamoxifen (n = 15). Scale bars, 100µm.

d. Hematoxylin and eosin staining of ileal tissue from 3-month old G0, G4, G0+TAM and G4+TAM mice. (n= 15) Scale bar, 100µm.

e. Hematoxylin and eosin staining of small intestinal tissue from 8-month old G0 and G4 treated with or without tamoxifen. (n= 15) Scale bars, 100µm.

f. Hematoxylin and eosin staining of colon tissue from 8-month old G4 mice (n = 15). Scale bars, 100µm. Arrows indicate cryptitis and crypt abscess.

g. Hematoxylin and eosin staining of colon tissue from 6-month old G4 mice treated with tamoxifen for 1month (n = 15). Scale bars, 100µm.

h. Hematoxylin and eosin staining of colon tissue from 6-month old G4 mice treated with tamoxifen for 2 months (n = 15). Scale bars, 100µm.

i. Histogram depicting the pathological scores of G0 and G4 mice treated with or without tamoxifen for 1 or 2 months. *P* values were calculated using two sided t test between the two groups as indicated. The following are the *p* values between G0 and G4, $p<0.0001$, between G4 and G4+TAM (1 month), $p=0.0120$, between G4 and G4+TAM (2 month), $p=0.0010$.

*statistically significant, $p<0.05$ by unpaired Student's t test, two tailed. n represents number of mice used in the study**.** Data are represented as mean ±SEM.

Supplementary Figure 2

Telomerase reactivation suppresses intestinal inflammation through epithelial cell intrinsic mechanism

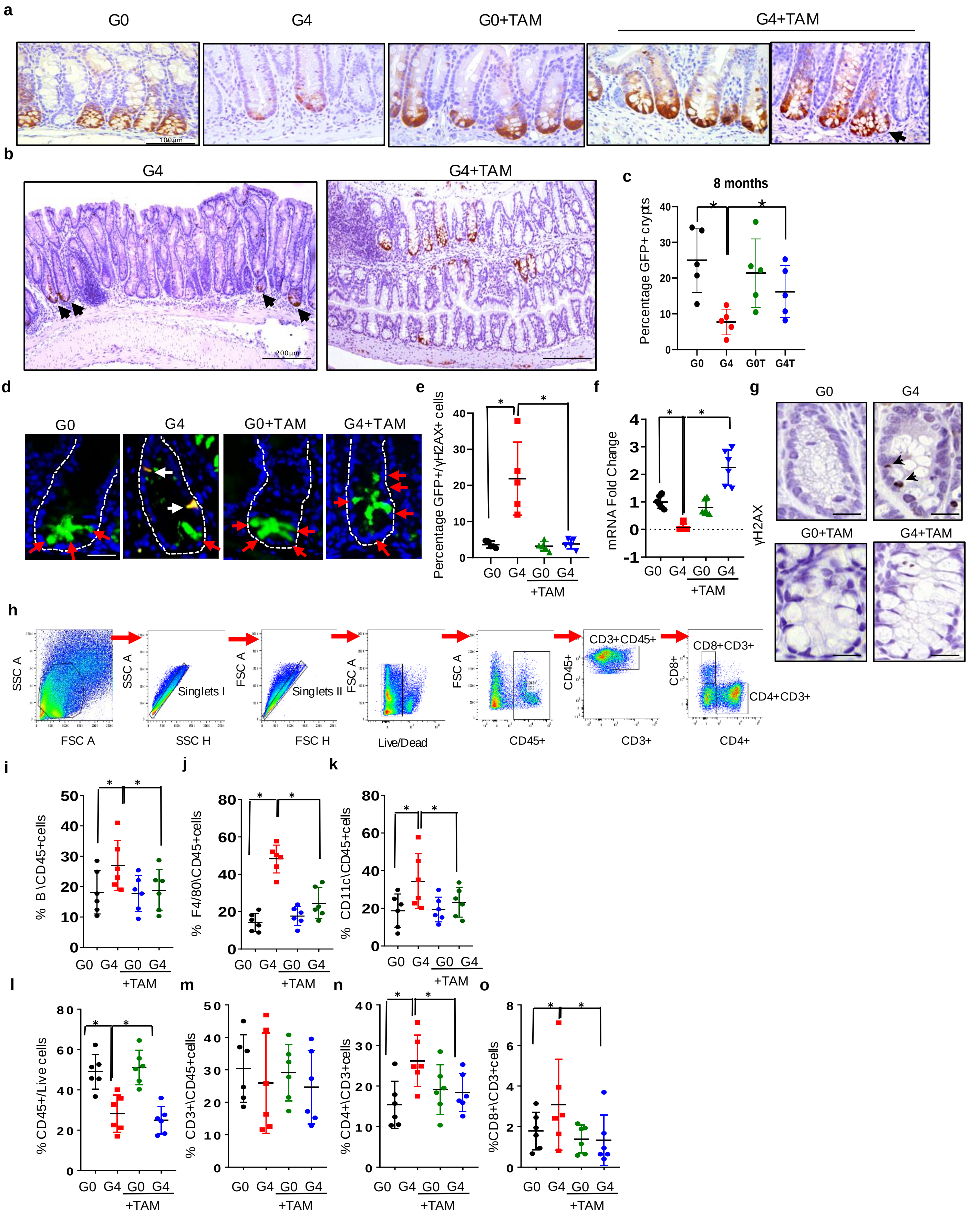

Supplementary Figure 2. Telomerase reactivation suppresses intestinal inflammation through epithelial cell intrinsic mechanisms

a. Immunostaining of G0 and G4 colonic crypts from 8-month old mice treated with or without tamoxifen with GFP. (n= 5). The black arrow denotes crypt fission. Magnification 100µm.

b. Immunostaining of G4 and G4+TAM colonic crypts from 8-month old mice. n = 5. Magnification 200µm.

c. Quantification of GFP positive crypts. (n = 5 mice). *P* values were calculated using two sided t test between G0 and G4, *p*= 0.0040 and between G4 and G4+TAM, *p*=0.0466.

d. Immunofluorescence with antibody for GFP and γH2AX in the crypts of G0, G4 mice treated with or without tamoxifen. (n = 5). White arrows denote GFP positive Lgr5 stem cells exhibiting damage (double positives, orange). Red arrows denote GFP positive Lgr5 stem cells (single positive, green). Scale bars, 50µm.

e. Quantification of 200 GFP+ crypts positive for γH2AX represented as percentage in the intestines of G0, G4 mice treated with or without tamoxifen (n = 5). *P* values were calculated using two sided t test between G0 and G4, *p*= 0.0038 and between G4 and G4 +TAM, *p*=0.0042.

f. qRT-PCR for telomerase RNA isolated from the crypts of mice of the indicated genotypes with or without tamoxifen treatment (n = 6). *P* values were calculated using two sided t test between G0 and G4, *p*= 0.0001 and between G4 and G4+TAM, *p*<0.0001.

g. Immunostaining of G0 and G4 colonic crypts from 3-month old mice treated with or without tamoxifen with γH2AX. (n= 5) Black arrows indicate positively stained cells. Scale bars, 50µm.

h. Gating strategy for flow cytometry.

i-k. Flow cytometric analysis of B cells, macrophages and dendritic cells from the lamina propria and intestinal epithelia from 8-month old mice of the indicated genotypes with or without tamoxifen treatment. (n = 6). *P* values were calculated using two sided t test between G0 and G4, G4 and G4+TAM. **i.** B220+, *p* value between G0 and G4, *p*=0.0774, between G4 and G4=TAM, *p*=0.0933. **j.** F4/80, *p* value between G0 and G4, *p*=0.0001, between G4 and G4+TAM, *p*=.0004.**k.** cd11c+, *p* value between G0 and G4, *p*=0.0495, between G4 and G4+TAM, *p*=0.1309.

l-o. Flow cytometric analysis of the indicated cell types from the lamina propria and intestinal epithelia from 3-month old mice of the indicated genotypes with or without tamoxifen treatment. (n = 6). *P* values were calculated using two sided t test between G0 and G4, G4 and G4+TAM. **l.** CD45+, *p* value between G0 and G4, *p*=0.0024, between G4 and G4=TAM, *p*=0.4924. **m.** CD3+, *p* value between G0 and G4, *p*=0.5651, between G4 and G4+TAM, *p*=0.8724.**k.** CD4+, *p* value between G0 and G4, *p*=0.0114, between G4 and G4+TAM, *p*=0.0364.**i.** CD8+, *p* value between G0 and G4, *p* =0.0203, between G4 and G4+TAM, *p*=0.0124.

*statistically significant, *p* < 0.05 by unpaired Student's t test, two -tailed. n represents number of mice used in the study. Each experiment was conducted at least two times. Data are represented as mean ±SEM.

Supplementary Figure 3

Identification of Yap1 as a key regulator of immune pathways including *IL18*

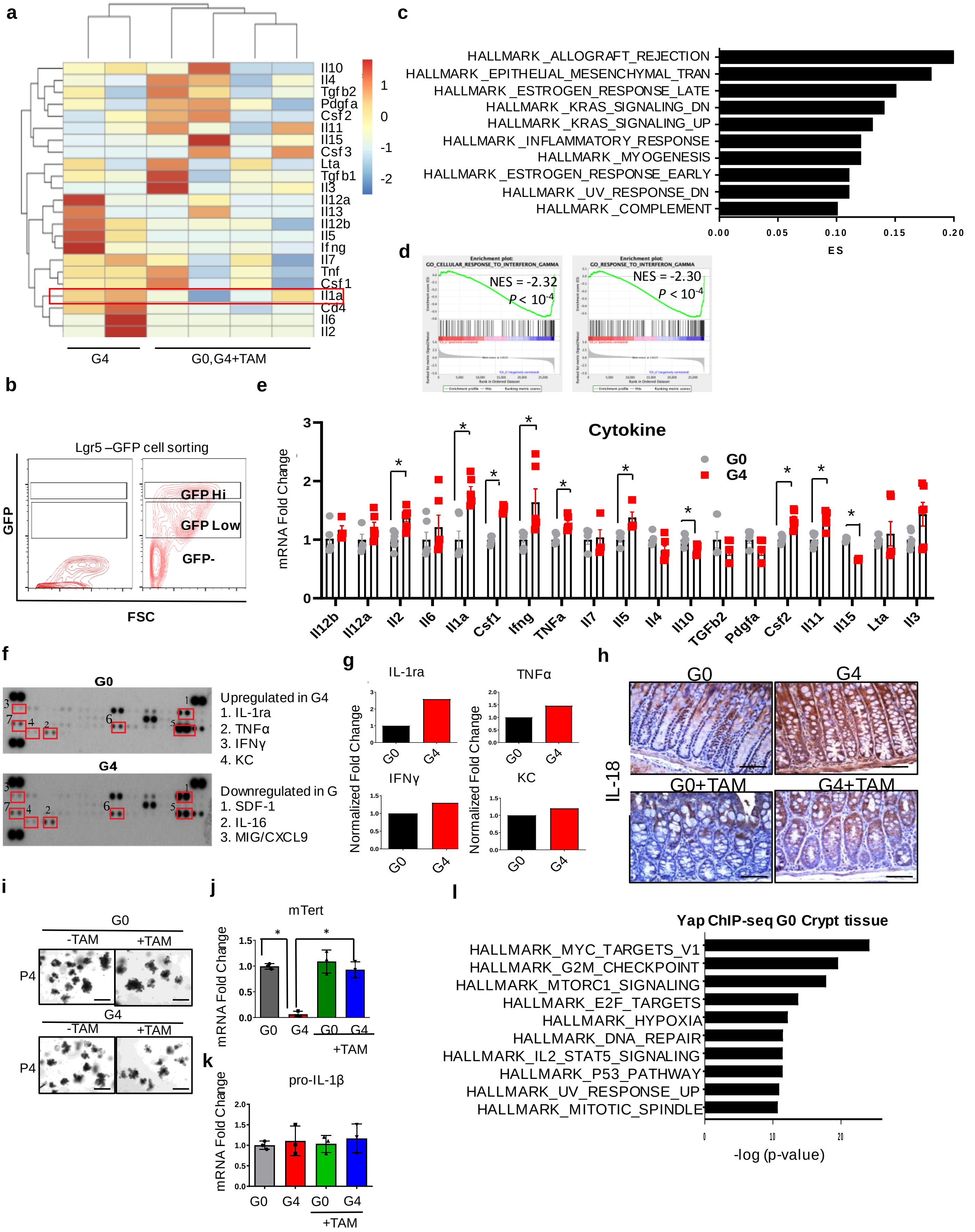

Supplementary Figure 3. Identification of YAP1 as a key regulator of immune pathways including *IL-18*

a. Heat map denoting the RNA-seq analysis performed on G0, G4 and G4+TAM mouse colonic crypts depicting a rescue in the inflammatory pathway genes in G4 mice. The red rectangle highlights the IL-1α pathway (n = 2). Red denotes high and blue denotes low in the scalbar.

b. Lgr5-GFP$^+$ cell sorting strategy, as sorted from the G0 and G4 mouse colons. Two distinct populations, GFP^{hi} and GFP^{low}, are discriminated. FSC, forward scatter.

c. Significantly deregulated pathways identified by GSEA pathway analysis of the RNA-seq from GFP+ colonic stem/progenitor sorted cells comparing G0 to G4 mice.

d. GSEA graphs for GO_CELLULAR_RESPONSE_TO_INTERFERON_GAMMA pathway, NES=-2.32, $p<10^4$ and GO_RESPONSE_TO_INTERFERON_GAMMA, NES-2.30, $p<10^4$

e. qRT-PCR with G0 and G4 enteroid RNA for the indicated cytokines as validation of the RNA-seq. (n=3) *P* values were calculated using multiple t tests, using the two stage linear step-up procedure of Banjamini, Krieger and Yekutieli, with Q=1%. The significant *P* values are specified between G0 and G4, for IL2, *p*=0.0587, Il1a, *p*=0.0410, Csf1, *p*=0.0008, IFNγ, *p*=0.0161, TNFα, *p*=0.0491, IL-10, *p*=0.0468, Csf2, *p*=0.0175, IL11, *p*=0.0102, IL15, *p*=0.00005.

f. Cytokine array performed on lysates from the colonic epithelial cells from G0 and G4 mice (n = 3).

g. Densitometric analysis of the cytokine dot blot shown in panel (e). The blot was quantified, and histograms are plotted for the top 4 cytokines that were downregulated or upregulated. (n= 3)

h. Immunohistochemistry for IL-18 in the colonic crypts from mice of the indicated genotypes (n = 3). Scale bar, 50µm.

i. Crypts were isolated from the ileum of G0 and G4 mice and cultured for 5 days with or without tamoxifen and subsequently passaged. Five-day old enteroid cultures after undergoing passaging 4 times are shown. Scale bars, 200µm.

j. qRT-PCR for telomerase RNA isolated from the crypts of mice of the indicated genotypes with or without tamoxifen treatment (n = 3). *P* values were calculated using two sided t test between G0 and G4, *p*= 0.0001 and between G4 and G4+TAM, *p*=0.0008.

k. qRT-PCR for pro-IL-1β with RNA isolated from the intestinal organoids of mice of the indicated genotypes with or without tamoxifen treatment (n = 3). *P* values were calculated using two sided t test

l. Gene enrichment analysis for the differentially bound genes by YAP1 in the G0 epithelium.

*statistically significant, *p*<0.05 by unpaired student's t test, two tailed and Fisher's Exact Test. n represents number of mice or patient biopsies used in the study**.** Each experiment was conducted at least two times**.** Data are represented as mean ±SEM.

Supplementary Figure 4

Telomere dysfunction activates YAP1

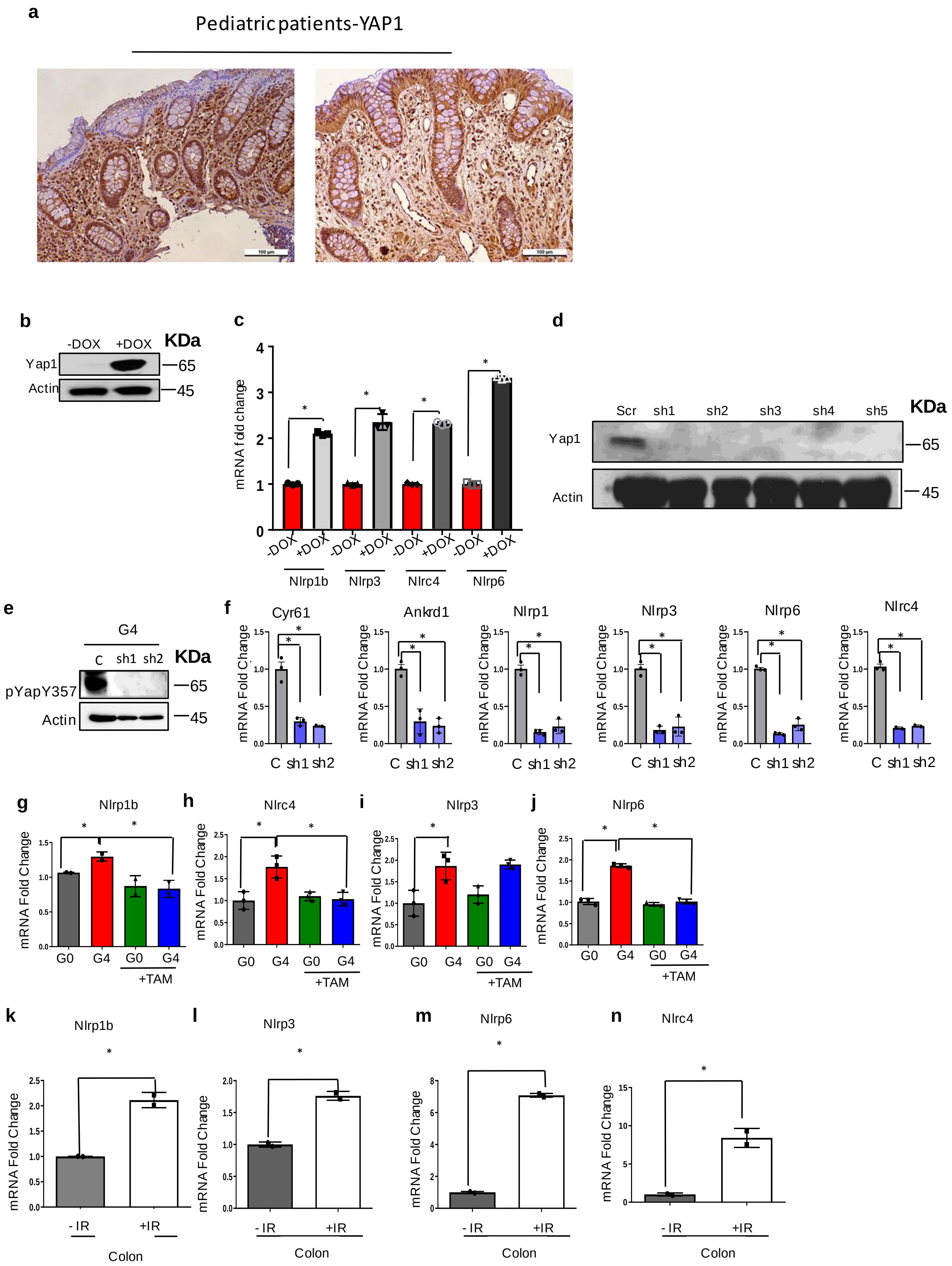

Supplementary Figure 4. Telomere dysfunction activates YAP1

a. Immunostaining for YAP1 in telomere dysfunctional pediatric patient biopsy samples. (n=3)**.**

b. Western blot for YAP1 of enteroid lysates from YAP1(S127A) transgenic model treated with or without doxycycline. (n = 3).

c. qRT-PCR of RNA from YAP1(S127A) transgenic mouse enteroids treated with or without doxycycline for the indicated genes (n = 3). *P* values were calculated using two sided t test between dox untreated and dox treated organoids. The following are the *p*-values for Nlrp1b, *p*<.0001, Nlrp3, *p*=0.0002, Nlrc4, *p*<0.0001 and Nlrp6, *p*<0.0001.

d. Western blot for YAP1 from the MC-38 cell line treated with control or lentiviral shRNAs against *Yap1*.

e. Western blot for pYAP(Y357) with lysates from control vector transduced or *YAP1* shRNAs transduced G4 enteroids (n = 2).

f. qRT-PCR analysis of the indicated genes from G4 enteroids transduced with control vector or *YAP1* shRNAs (n= 3). *P* value was calculated with t test, two sided. *p* value between control and YAPsh1, *p*=0.0021, and control and YAPsh2, *p*=0.0082 (Cyr61), *p* value between control and YAPsh1, *p*=0.0032, and control and YAPsh2, *p*=0.0007 (Ankrd1), *p* value between control and YAPsh1, *p*<0.0001, and control and YAPsh2, *p*=0.0005 (Nlrp1b), *p* value between control and YAPsh1, *p*=0.0002, and control and YAPsh2, *p*=0.0011 (Nlrp3), *p* value between control and YAPsh1, *p*<0.0001, and control and YAPsh2, *p*=0.0001 (Nlrp6), *p* value between control and YAPsh1, *p*=0.0004, and control and YAPsh2, *p*=0.0004 (Nlrc4).

g-j. qRT-PCR analysis of the inflammasome pathway genes from enteroids derived from the mice of the indicated genotypes. **(g)** *Nlrp1b*, *p* value between G0 and G4, *p*=0.0383, between G4 and G4=TAM,p=0.0422(**h)** *Nlrc4*, *p* value between G0 and G4*,* *p*=0.0151, between G4 and G4=TAM,*p*=0.0133 **(i)** *Nlrp3*, *p* value between G0 and G4, *p*=0.0276, between G4 and G4=TAM,*p*=0.8766 **(j)** *Nlrp6, p* value between G0 and G4, *p*<0.0001, between G4 and G4=TAM,*p*<0.0001. (n=3 for each).

k-n. qRT-PCR of RNA from control or irradiated colonic G0 mouse crypts for **(k)** Nlrp1b,*p*=0.0089 **(l)** Nlrp3, *p*=0.0052 **(m)** Nlrp6,*p*=0.0002 **(n)** Nlrc4, *p*=0.0141 (n = 3). *P* value was calculated with t test, two sided.

*statistically significant, *p*<0.05 by unpaired Student's t test, two tailed. n represents number of mice used in the study**.** Each experiment was conducted at least two times. Data are represented as mean ±SEM.

Supplementary Figure 5

Pharmacologic inhibition of ATM, YAP1 and caspase-1 *in vitro* and *in vivo* reduces IL-18 secretion and ameliorates inflammation

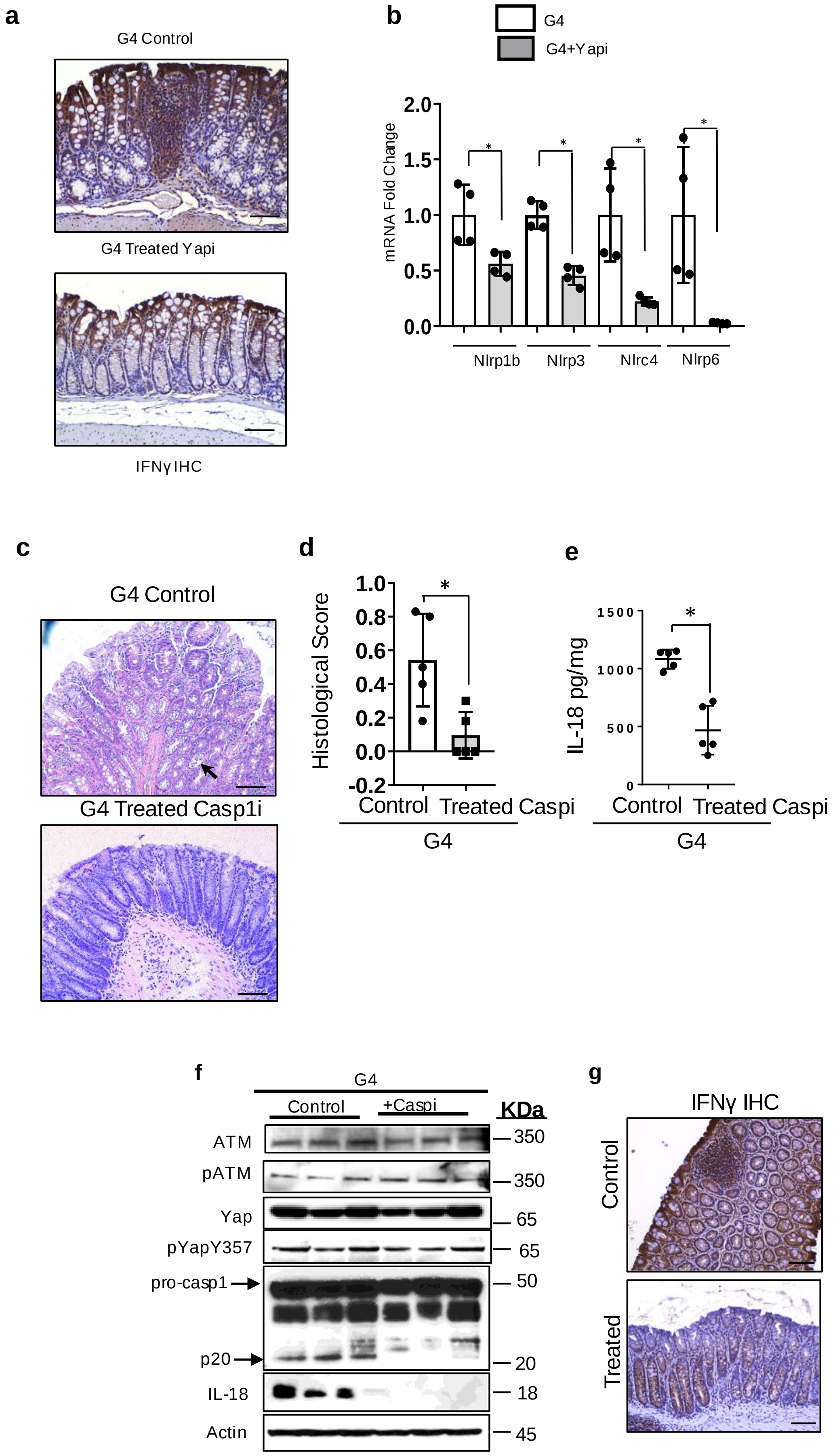

Supplementary Figure 5. Pharmacologic inhibition of ATM, YAP1 and caspase-1 *in vitro* and *in vivo* reduces IL-18 secretion and ameliorates inflammation.

a. Immunohistochemistry for IFN-γ with tissue from G4 mice treated with or without the YAP1 inhibitor verteporfin (n = 3).

b. qRT-PCR analysis of the indicated inflammasome pathway genes from the colonic intestinal tissue lysate of mice treated with or without YAP1 inhibitor (n = 4). *P* values were calculated using two sided t test and are as specified, Nlrp1b, *p*=0.0238, Nlrp3, *p*=0.0003, Nlrc4, *p*=0.0100 and Nlrp6, *p*=0.0188.

c. Hematoxylin and eosin micrographs from colonic epithelium of G4 mice treated with or without the caspase-1 inhibitor, Ac-YVAD-cmk (casp1i), (n = 6). Scale bars, 50μm.

d. Histopathological score of the intestinal phenotype of the G4 mice treated with or without Ac-YVAD-cmk (casp1i) (n = 6). *P* values were calculated using two sided t test, *p*=0.0119.

e. ELISA for the quantification of mature IL-18 from the colonic epithelium of the G4 mice treated with or without Ac-YVAD-cmk (casp1i) (n = 6). *P* values were calculated using two sided t test, *p*=0.0003.

f. Western blot with colonic lysate from the colonic epithelium of the G4 mice treated with or without Ac-YVAD-cmk (casp1i) for the indicated antibodies (n = 3).

g. Immunohistochemistry for IFN-γ with tissue from G4 mice treated with or without caspase-1 inhibitor (n = 3).

*statistically significant, *p*<0.05 by unpaired Student's t test. n represents number of mice used in the study. Data are represented as mean ±SEM. Experiments were conducted at least 2 independent times.

Supplementary Figure 6

Antibiotic treatment of the telomere dysfunctional mice reduces IL18 secretion and ameliorates inflammation

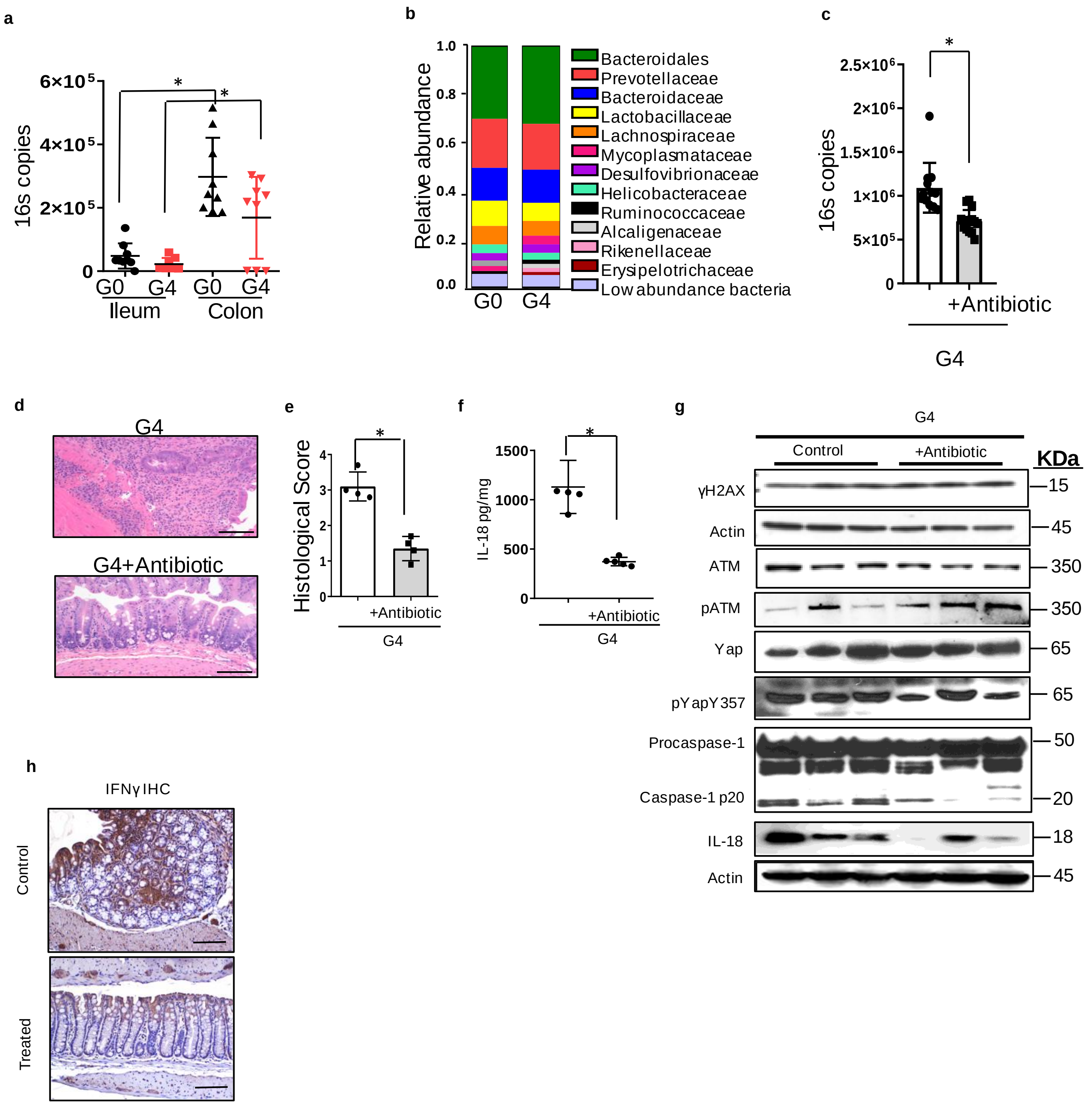

Supplementary Figure 6. Antibiotic treatment of telomere dysfunctional mice reduces IL-18 secretion and ameliorates inflammation

a. qRT-PCR for bacterial 16S rDNA with luminal content from ileum (I) and fecal content from the colon (C) of G0 and G4 mice at 3 months of age (G0-I, n = 9, G4-I, n= 8, G0-C, n= 9, G4-C, n= 9). *P* values were calculated using two sided t test. G0-I compared to G0-C, $p<0.0001$ and G4-I compared to G4-C, $p=0.0065$

b. Average relative abundance of bacterial genera that represents >1% abundance in the fecal colon microbiota of G0 and G4 mice at both 3 months and 6 months of age (n = 6).

c. Bacterial load enumeration by quantification of the 16s copy numbers from the mice of the indicated genotypes demonstrating reduction in load after treatment with antibiotics (G0, n = 12, G4, n= 13). *P* values were calculated using two sided t test, $p<0.0001$.

d. Hematoxylin and eosin staining of colon tissue from 5-6-month-old G4 mice treated with or without antibiotic (TMS) in water (n = 5). Scale bars, 50µm.

e. Histological scoring of the large intestinal lesions based on criteria stated in "Methods" from antibiotic treated and untreated G4 mice.(n= 4). *P* values were calculated using two sided t test, $p=0.0006$.

f. ELISA for IL-18 with the colonic tissue lysates of G4 mice treated with or without antibiotics (n = 5). *P* values were calculated using two sided t test, $p=0.0003$

g. Western blot of colonic lysate from the colonic epithelium of the G4 mice treated with or without antibiotics for the indicated antibodies (n = 3).

h. Immunohistochemistry for IFN-γ with tissue from G4 mice treated with or without antibiotic (n = 3).

*statistically significant, $p<0.05$ by unpaired Student's t test, two tailed. n represents number of mice used in the study. Data are represented as mean ±SEM.

Supplementary Table 1: Pathological colitis severity

Genotype	Age	n	Ileal disease score (0-5)	Colonic disease score (0-5)
**G0**	3mo	15	0	0
	8mo	15	0.05±0.008	0.1±0.02
**G4**	3mo	15	0.1±0.02	0.53±0.13
	8mo	15	0.56±0.03	3.2±0.15
**G0+TAM**	3mo	15	0	0
	8mo	10	0.08±0.1	0.11 ± 0.025
**G4+TAM**	3mo	15	0.20±0.10	0.73±0.18
	8mo	15	0.07±0.02	1.16±0.13

Supplementary Table 2: Clinical characteristics of telomere biology disorder study subjects with GI disease

Case	Genetic Diagnosis	Clinical features associated with telomere biology disorder	Age at time of GI biopsy	GI findings
1	*ACD*<br>NM_0010824861<br>c.505-507delGAG /c.619delG<br>(TPP1p.E169del/ p.D207Tfs*22)	T+,B-, NK- immunodeficiency<br>Microcephaly	2 y 5 m	Chronic gastritis, acute enteritis at the ileocecal valve, pancolitis with increased eosinophils with evidence of chronicity.
2	*TINF2*<br>NM_001099274<br>c.805C>T<br>(TIN2p.Q269*)	Bone marrow failure, s/p bone marrow transplantation (age 4 y 11 m)<br>Mucocutaneous triad<br>Gastrointestinal bleeding<br>Pulmonary fibrosis	10 y	Esophagitis, mild chronic inflammation in the stomach, chronic inflammation in the lamina propria of the small and large intestine, increased crypt apoptosis.
3	*TINF2*<br>NM_001099274<br>c.811C>T<br>(TIN2p.Q271*)	Bone marrow failure, s/p bone marrow transplantation (3 yr 9 m)<br>Mucocutaneous triad<br>Hepatopulmonary syndrome	11 y	Chronic inflammation in stomach and duodenum.
4	*TINF2*<br>NM_001099274<br>c.845G>A<br>(TIN2p.R282H)	Bone marrow failure, s/p bone marrow transplantation (5 y 2 m)<br>Mucocutaneous triad<br>Pulmonary fibrosis<br>Portal hypertension	23 y	Mild chronic reflux esophagitis, chronic inflammation in the stomach, mild lymphoplasmacytic inflammation in the small and the large intestine, acute colitis, increased scattered apoptosis.
5	*TERT*<br>NM_198253<br>c.2110C>T<br>(TERTp.704S)	Increased toxicity to chemotherapy for Hodgkin lymphoma<br>Bone marrow hypocellularity<br>Nail dysplasia	17 yrs	Reactive epithelial changes in the esophagus suggestive of chronic reflux, lymphoplasmacytic infiltrates with significant intra-epithelial T cell infiltrate in the duodenum, lymphonodular hyperplasia in the ascending colon.

Supplementary Table 3: qRT-PCR primer sequences

Gene Name	Sequence
mTert (mouse)	5'-GCACTTTGGTTGCCCAATG-3'F
	5'-GCACGTTTCTCTCGTTGCG-3'R
pro-IL-1β (mouse)	5'-CTCCATGAGCTTTGTACAAGG-3'F
	5'-TGCTGATGTACCAGTTGGGG-3'R
pro-IL-18 (mouse)	5'-ACTGTACAACCGCAGTAATACGG-3'F
	5'- AGTGAACATTACAGATTTATCCC-3'R
Nlrc4 (mouse)	5'-CTACATTGATGCTGCCTTGG-3'R
	5'-ATCCGTCACTGCTCACACAG-3'R
Nlrp3 (mouse)	5'- ATTACCCGCCCGAGAAAGG-3'F
	5'- CATGAGTGTGGCTAGATCCAAG-3'R
Nlrp1b (mouse)	5'-CACTGCCCAAGATTGCTACA-3'F
	5'-CTTCACTCAGCACCAGACCA-3'R
Nlrp6 (mouse)	5'-CACACCCAGAATGAGACCAG-3'F
	5'-GTAGCCATAAGCAGCTCCCT-3'R
Vgll3 (mouse)	5'-CCGGAACCCCTGGCAG-3'F
	5'-CTTGTCCTGATGCTGAAGACC-3'R
Ankrd1 (mouse)	5'-GGAACAACGGAAAAGCGAGAA-3'F
	5'-GAAACCTCGGCACATCCACA-3'R
Cyr61 (mouse)	5'-CAGCTCACTGAAGAGGCTTC-3'F
	5'-GCGTGCAGAGGGTTGAAAAG-3'R
Yap1 (mouse)	5'-ACCCTCGTTTTGCCATGAAC-3'F
	5'-TGTGCTGGGATTGATATTCCGTA-3'R
Csf1 (mouse)	5'-ATGAGCAGGAGTATTGCCAAGG-3'F
	5'-TCCATTCCCAATCATGTGGCTA-3'R
IL-11 (mouse)	5'-TGTTCTCCTAACCCGATCCCT-3'F
	5'-CAGGAAGCTGCAAAGATCCCA-3'R
IL-2 (mouse)	5'-CCTGAGCAGGGAGAATTACA-3'R
	5'-TCCAGAACATGCCGCAGA-3'R
CD4 (mouse)	5'-GAGAGTCAGCGGAGTTCTC-3'R
	5'-CTCACAGGTCAAAGTATTGTTG-3'R
IL-7 (mouse)	5'- TCTGCTGCCTGTCACATCATC-3'F
	5'- GGACATTGAATTCTTCACTGATATTCA-3'R
IFNg (mouse)	5'-TGAACGCTACACACTGCATCTTGG-3'F
	5'-CGACTCCTTTTCCGCTTCCTGAG-3'R
IL-5 (mouse)	5'-TCACCGAGCTCTGTTGACAA-3'F
	5'-CCACACTTCTCTTTTTGGCG-3'R
IL-12b (mouse)	5'-GCACATCAGACCAGGCAGC-3'F
	5'-CATGCCCACTTGCTGCATGA-3'R
IL-12a (mouse)	5'-CCAGGTGTCTTAGCCAGTC-3'F
	5'-CTCGTTCTTGTGTAGTTCCAG-3'R
IL-13 (mouse)	5'-TGAGGAGCTGAGCAACATCACACA-3'F
	5'-TGCGGTTACAGAGGCCATGCAATA-3'R
IL-10 (mouse)	5'-ATTTGAATTCCCTGGGTGAGAAG-3'F
	5'-CACAGGGGAGAAATCGATGACA-3'R
IL-4 (mouse)	5'-TCGGCATTTTGAACGAGGTC-3'F
	5'-GAAAAGCCCGAAAGAGTCTC-3'R

TGFb1 (mouse)	5’-AGCAGTGCCCGAACCCCCAT-3’F
	5’-GGGGTCAGCAGCCGGTTACC-3’R
TGFb2 (mouse)	5’-CAGGAGTGGCTTCACCACAAAG-3’R
	5’-TGGCATATGTAGAGGTGCCATCA-3’R
PDGFa (mouse)	5’-CTCTTGGAGATAGACTCCGTAGG-3’R
	5’-ACTTCTCTTCCTGCGAATGG-3’R
Csf3 (mouse)	5’- TCTGCTGCCTGTCACATCATC-3’F
	5’- GGAAGGGAGACCAGATGCT-3’R
Csf2 (mouse)	5’-ATGCCTGTCACGTTGAATGAAG-3’F
	5’-GCGGGTCTGCACACATGTTA-3’R
IL-15 (mouse)	5’-GGAATCCAACTGGATAGATGTAAGATA-3’F
	5’-TGCTCGAGGGACGTGTTGATGAACAT-3’R
Lta (mouse)	5’-CACGAGGTCCAGCTCTTTTC-3’F
	5’-AGTGCAAAGGCTCCAAAGAA-3’R
IL-1a (mouse)	5’-ATCAGTACCTCACGGCTGCT-3’R
	5’-TGGGTATCTCAGGCATCTCC-3’R
Il-6 (mouse)	5’-CTCTGGGAAATCGTGGAAAT-3’R
	5’-CCAGTTTGGTAGCATCCATC-3’R
TNFa (mouse)	5’-ATGAGAAGTICCCAAATGGC-3’R
	5’-CTCCACTTGGTGGTTTGCTA-3’R
IL-3 (mouse)	5’-CTGCCTACATCTGCGAATGACT-3’F
	5’-CAGATCGTTAAGGTGGACCATG-3’R
IL-18 (mouse)	5’-GCTTGAATCTAAATTATCAGTC-3’F
	5’-GAAGATTCAAATTGCATCTTAT-3’R
GAPDH (mouse)	5’-AGGTCGGTGTGAACGGATTTG-3’F
	5’-TGTAGACCATGTAGTTGAGGTCA-3’R

Supplementary Table 4 : List of lentiviral shRNA plasmids used in the study

TRC ID	Gene	Comments
	YAP1	
TRCN0000238432(sh1)		Used in the assays
TRCN0000238434(sh2)		Used in the assays
TRCN0000238436		
TRCN0000095864		
TRCN0000095866		
