# Supplementary material for: Telomere dysfunction activates YAP1 to drive tissue inflammation
Source: Nat Commun. 2020 Sep 21;11:4766. doi: 10.1038/s41467-020-18420-w (PMC7505960; doi:10.1038/s41467-020-18420-w)
Supplement: Supplementary file 2 — Reporting Summary [file 41467_2020_18420_MOESM2_ESM.pdf]

nature research

Corresponding author(s): Ronald DePinho

Last updated by author(s): July 29, 2020

Reporting Summary

Nature Research wishes to improve the reproducibility of the work that we publish. This form provides structure for consistency and transparency in reporting. For further information on Nature Research policies, see Authors & Referees and the Editorial Policy Checklist.

Statistics

For all statistical analyses, confirm that the following items are present in the figure legend, table legend, main text, or Methods section.

n/a	Confirmed	
☐	☒	The exact sample size (*n*) for each experimental group/condition, given as a discrete number and unit of measurement
☐	☒	A statement on whether measurements were taken from distinct samples or whether the same sample was measured repeatedly
☐	☒	The statistical test(s) used AND whether they are one- or two-sided<br>*Only common tests should be described solely by name; describe more complex techniques in the Methods section.*
☐	☒	A description of all covariates tested
☐	☒	A description of any assumptions or corrections, such as tests of normality and adjustment for multiple comparisons
☐	☒	A full description of the statistical parameters including central tendency (e.g. means) or other basic estimates (e.g. regression coefficient) AND variation (e.g. standard deviation) or associated estimates of uncertainty (e.g. confidence intervals)
☐	X	For null hypothesis testing, the test statistic (e.g. *F*, *t*, *r*) with confidence intervals, effect sizes, degrees of freedom and *P* value noted<br>*Give P values as exact values whenever suitable.*
☒	☐	For Bayesian analysis, information on the choice of priors and Markov chain Monte Carlo settings
☒	☐	For hierarchical and complex designs, identification of the appropriate level for tests and full reporting of outcomes
☐	☒	Estimates of effect sizes (e.g. Cohen's *d*, Pearson's *r*), indicating how they were calculated

Our web collection on statistics for biologists contains articles on many of the points above.

Software and code

Policy information about availability of computer code

Data collection

No software was used for data collection.

Data analysis

Raw sequencing data (BCL format) were converted to Fastq files using Illumina CASAVA software (v1.8.2) and aligned to the mouse reference genome (mm10) using STAR software 74. The HTSeq-count program was used to generate raw read counts for each gene 75. The R package edgeR 76 v3.14.0 was used for data normalization and differential expression analysis using the criteria of log2 fold change ≥ 0.5 or ≤ -0.5 and p-value ≤ 0.1. Pathway enrichment analysis was performed using GSEA software 77 based on p-value from the aforementioned differential expression analysis, and clustering visualization was done by the R package heat map.
Microbiome community studies were performed at the Alkek Center for Metagenomics and Microbiome Research at Baylor College of Medicine, Houston, Texas. DNA was extracted from fecal pellets, and the 16S rRNA V4 region was amplified by PCR. Products were sequenced using an Illumina MiSeq platform and a 2x250 bp paired-end protocol as described previously 80,81. 16S rRNA gene sequences were assigned into operational taxonomic units (OTUs) using the UPARSE pipeline and alignment to the SILVA SSURef_NR99_119 database at 97% sequence identity 82. Analysis and visualization of microbiome communities were conducted with the publicly available software R (R Core Team 2015, version 3.2.2), utilizing the phyloseq package (Bioconductor) to import sample data, calculate α- and β-diversity metrics, and microbiome community profiles 83,84. For the 16S rRNA gene quantification, bacterial DNA was extracted by a previously described method using the MO BIO PowerSoil DNA Isolation Kit (MO BIO Laboratories) following the manufacturer's instructions and quantified using Qubit (Life Technologies) 85. Quantitative PCR was performed in QuantStudio 7 Real-Time PCR System using PerfeCTa SYBR Green Fast Mix. The qPCR primers (1369F-1492R) target regions flanking V9 of the 16S rRNA gene. A standard curve was made using a serially diluted plasmid that contained nt 1369 to 1492 of an E. coli 16S rRNA gene. The concentrations of unknowns were calculated from CT values using the equation generated from plotting the standard curve. All samples

were run in triplicate, including the standard curve, a set of non-template controls (NTC), and inhibitor controls (known positives + unknown DNA).
Raw fastq reads for all ChIP-seq experiments were processed using FastQC (http://www.bioinformatics.babraham.ac.uk/projects/fastqc/), and quality reads were aligned to the mm9 reference genome using Bowtie version 1.2.251 with the following criteria: --best --chunkmbs 320. To directly compare G0 and G3 ChIP-seq samples, uniquely mapped reads for each mark were normalized by total reads per condition, sorted, and indexed using samtools version 1.953.
Model-based analysis of ChIP-seq (MACS) (version 1.4.2)54 was used to identify YAP1 enrichment over "input" background. MACS2 was used to identify the differential binding of YAP1 in G0 and G3 with the following criteria: bdgdiff –g 60 –l 120.To visualize ChIP-seq libraries on the IGV browser, we used deepTools version 2.7.15 to generate bigWig files by scaling the bam files to reads per kilobase per million (RPKM) using the following criteria: bamCoverage –b–normalizeUsing RPKM–smoothLength 300–binSize 30–extendReads 200 –o.
Image J

For manuscripts utilizing custom algorithms or software that are central to the research but not yet described in published literature, software must be made available to editors/reviewers. We strongly encourage code deposition in a community repository (e.g. GitHub). See the Nature Research guidelines for submitting code & software for further information.

Data

Policy information about availability of data

All manuscripts must include a data availability statement. This statement should provide the following information, where applicable:

- Accession codes, unique identifiers, or web links for publicly available datasets
- A list of figures that have associated raw data
- A description of any restrictions on data availability

The GEO accession code for the RNA sequence data is GSE108902 and the GEO accession code for ChIP-seq data is GSE144200, To analyze YAP signature genes, the list of YAP signature genes (PMID: 26503053) is got and formatted as the compatible pathway database (grp format) with GSEA. GSEA analysis find that YAP signature genes are significantly up-expressed in our RNAseq expression data with NES (Normalized Enrichment Score) = 1.36 and FDR-corrected p-Value 6.45 x 10-3. Mouse reference genome mm10 was used for alignment.

Field-specific reporting

Please select the one below that is the best fit for your research. If you are not sure, read the appropriate sections before making your selection.

☒ Life sciences ☐ Behavioural & social sciences ☐ Ecological, evolutionary & environmental sciences

For a reference copy of the document with all sections, see nature.com/documents/nr-reporting-summary-flat.pdf

Life sciences study design

All studies must disclose on these points even when the disclosure is negative.

Sample size	A minimum sample size of 3 was used for all the in vitro experiments and a sample size of 5/6 for the in vivo experiments. Given the high penetrance of the phenotype based on our experience with the model, this sample size was sufficient to obtain a significant p value both *in vitro* and *in vivo*. 60 human sample biopsies were analyzed.
Data exclusions	No data were excluded from the analyses of samples.
Replication	All attempts at replicating the results were successful. Each experiment was successfully replicated atleast two independent times
Randomization	Mice from the same genotype were allocated to various groups (control or treated) randomly.
Blinding	Data analysis by the pathologist was done in a blinded manner.

Reporting for specific materials, systems and methods

We require information from authors about some types of materials, experimental systems and methods used in many studies. Here, indicate whether each material, system or method listed is relevant to your study. If you are not sure if a list item applies to your research, read the appropriate section before selecting a response.

Materials & experimental systems

n/a	Involved in the study	
☐	☒	Antibodies
☐	☒	Eukaryotic cell lines
☒	☐	Palaeontology
☐	☒	Animals and other organisms
☒	☐	Human research participants
☒	☐	Clinical data

Methods

n/a	Involved in the study	
☐	☒	ChIP-seq
☐	☒	Flow cytometry
☒	☐	MRI-based neuroimaging

Antibodies

Antibodies used

The following antibodies were used for western blot: GFP (CST, catalogue#2956, Abcam, catalogue# ab13970, 1:200), caspase-1 (p20), Adipogen, catalogue #AG-20B-0042-C100, 1:1000), anti-phospho-histone H2AX (Ser139), Milipore, clone JBW301, catalogue#05-636, 1:1000, H2AX, (CST, D17A3, 1:1000, catalogue #2595), pre-IL-18 antibody, Proteintech , catalogue#60070-1-Ig, CloneNo.: 5C6F8,1:1000, mature IL18, MBL International corporation, catalogue #D046-3,clone 39-3F, 1:1000, ATM antibody, Novus Biologicals, clone 2C1, catalogue#NB100-104,1:1000, pATM (Ser1981), Novus biologicals, clone 10H11.E12, catalogue#NB100-306, clone#10H11.E12,1:1000, c-Abl antibody, Cell Signaling Technologies, catalogue #2862,1:1000, Chk2 Antibody, Cell Signaling Technologies, catalogue##2662,1:1000, pChk2 (Thr68), Cell Signaling technologies, catalogue#2661,1:1000, Yap1. Novus Biologicals, catalogue#NB110-58358,1:1000, pYapY357, Abcam, catalogue#,ab62751,1:1000, NF-kB, Cell Signaling technologies, catalogue#8242S, clone#D14E12,1:1000, Phospho-NF-κB p65 (Ser536), Cell Signaling technologies,
catalogue#3033, clone#93H1,1:1000, IkB,Cell Signaling technologies, catalogue#9242,1:1000, Phospho-IκBα (Ser32/36), Cell signaling technologies, catalogue #9246,1:1000, clone#5A5, TRF2, Novus Biologicals, catalogue#NB110-57130, 1:1000, cleaved caspase-3 (Asp175), cell signaling technologies,catalogue#9664, clone#5A1E, 1:1000, Actin, Millipore Sigma, catalogue # A5441,clone#clone AC-15,1:1000 .
For immunohistochemistry and immunofluorescnece the following antibodies were used: IL-18 , Sigmaaldrich, catalogue#HPA003980,1:200, Anti-Interferon gamma antibody, abcam, catalogue#ab9657,1:200, GFP, Abcam, catalogue #,ab13970, 1:500.
For flowcytometric analysis the following antibodies were used in the dilutio of 1:20, from Biolegend, APC/Cy7 anti-mouse CD45 Antibody,catalogue#103115 ,Clone#30-F11, Brilliant Violet 510™ anti-mouse CD3 Antibody, catalogue#100233, clone# 17A2, PE/Cy7 anti-mouse CD4 Antibody, catalogue#100421, clone#GK1.5, Alexa Fluor® 700 anti-mouse CD8a Antibody, atalogue#100729, clone#53-6.7, Brilliant Violet 605™ anti-mouse/human CD11b Antibody, catalogue#101257, clone M1/70, PE anti-mouse/human CD45R/B220 Antibody, catalogue#103207 , clone#RA3-6B2, PerCP anti-mouse F4/80 Antibody, catalogue#123125, clone#BM8.

Validation

We have attached a separate sheet with specifications for antibody validation as specified by the manufacturer, due to lack of space.

Eukaryotic cell lines

Policy information about cell lines

Cell line source(s)	CRL-1831 cell line has been used from ATCC.
Authentication	We did not authenticate the cell line.
Mycoplasma contamination	Cell line was tested and found to be mycoplasma free.
Commonly misidentified lines (See ICLAC register)	No such line was used for the study

Palaeontology

Specimen provenance	*Provide provenance information for specimens and describe permits that were obtained for the work (including the name of the issuing authority, the date of issue, and any identifying information).*
Specimen deposition	*Indicate where the specimens have been deposited to permit free access by other researchers.*
Dating methods	*If new dates are provided, describe how they were obtained (e.g. collection, storage, sample pretreatment and measurement), where they were obtained (i.e. lab name), the calibration program and the protocol for quality assurance OR state that no new dates are provided.*

☐ Tick this box to confirm that the raw and calibrated dates are available in the paper or in Supplementary Information.

Animals and other organisms

Policy information about studies involving animals; ARRIVE guidelines recommended for reporting animal research

Laboratory animals	Gentically engineered mice used in the study are C57/BL6 background. Both males and females were used for the study. Ages ranging from 1,3,6 and 8 month old animals were used. Survival curves were obtained from animals of varying ages
Wild animals	The study does not include any wild animals.
Field-collected samples	The study does not include any such samples.
Ethics oversight	Approval was granted by the IRB and IACUC of MD Anderson Cancer Center.

Note that full information on the approval of the study protocol must also be provided in the manuscript.

Human research participants

Policy information about studies involving human research participants

Population characteristics	*Describe the covariate-relevant population characteristics of the human research participants (e.g. age, gender, genotypic information, past and current diagnosis and treatment categories). If you filled out the behavioural & social sciences study design questions and have nothing to add here, write "See above."*
Recruitment	*Describe how participants were recruited. Outline any potential self-selection bias or other biases that may be present and how these are likely to impact results.*
Ethics oversight	*Identify the organization(s) that approved the study protocol.*

Note that full information on the approval of the study protocol must also be provided in the manuscript.

Clinical data

Policy information about clinical studies

All manuscripts should comply with the ICMJE guidelines for publication of clinical research and a completed CONSORT checklist must be included with all submissions.

Clinical trial registration	*Provide the trial registration number from ClinicalTrials.gov or an equivalent agency.*
Study protocol	*Note where the full trial protocol can be accessed OR if not available, explain why.*
Data collection	*Describe the settings and locales of data collection, noting the time periods of recruitment and data collection.*
Outcomes	*Describe how you pre-defined primary and secondary outcome measures and how you assessed these measures.*

ChIP-seq

Data deposition

☒ Confirm that both raw and final processed data have been deposited in a public database such as GEO.

☒ Confirm that you have deposited or provided access to graph files (e.g. BED files) for the called peaks.

Data access links<br>*May remain private before publication.*	GSE144200
Files in database submission	fastq and bigwig files
Genome browser session<br>(e.g. UCSC)	UCSC

Methodology

Replicates	ChIP-seq was performed on the colonic epithelial crypt cells from two different groups, G0 telomere proficient and G3 telomere deficient animals. 3 mice from each group were utilized to isolate the epithelial cells and further processing was done.
Sequencing depth	Sample name Total reads Unique reads<br>G0Ab 20973501 19914696<br>G3Ab 20937125 18742020<br>G0I 21267475 14888374<br>G3I 19057641 17596903<br>All reads are single-end reads with read length of 76bp.
Antibodies	YAP antibody was used from Novus Biologicals NB110-58358
Peak calling parameters	Model-based analysis of ChIP-seq (MACS) (version 1.4.2) was used to identify YAP1 enrichment over "input" background.<br>MACS commands are listed below:<br>macs14 -t G0Ab.sorted.bam -c G0I.sorted.bam -n G0 -g mm<br>macs14 -t G3Ab.sorted.bam -c G3I.sorted.bam -n G3 -g mm
Data quality	Sample name Peaks (>5% FDR, >3 fold change)<br>G0Ab 5192<br>G3Ab 13531
Software	Raw fastq reads for all ChIP-seq experiments were processed using FastQC (http://www.bioinformatics.babraham.ac.uk/projects/fastqc/), and quality reads were aligned to the mm9 reference genome using Bowtie version 1.2.2 with the following criteria: --best --chunkmbs 320. To directly compare G0 and G3 ChIP-seq samples, uniquely mapped reads for each

mark were normalized by total reads per condition, sorted, and indexed using samtools version 1.9. Model-based analysis of ChIP-seq (MACS) (version 1.4.2) was used to identify YAP1 enrichment over “input” background. MACS2 was used to identify the differential binding of YAP1 in G0 and G3 with the following criteria: bdgdiff –g 60 –l 120.To visualize ChIP-seq libraries on the IGV browser, we used deepTools version 2.7.15 to generate bigWig files by scaling the bam files to reads per kilobase per million (RPKM) using the following criteria: bamCoverage –b–normalizeUsing RPKM–smoothLength 300–binSize 30–extendReads 200 –o.

Flow Cytometry

Plots

Confirm that:

☒ The axis labels state the marker and fluorochrome used (e.g. CD4-FITC).

☒ The axis scales are clearly visible. Include numbers along axes only for bottom left plot of group (a 'group' is an analysis of identical markers).

☒ All plots are contour plots with outliers or pseudocolor plots.

☒ A numerical value for number of cells or percentage (with statistics) is provided.

Methodology

Sample preparation	The immune cells were collected from the colonic lamina propria of the mice. The entire colons were digested with collagenase VIII and DNAseI sequentially twice for 20 minutes at 37 degree centigrade and then filtered first through .45 micron filter and subsequently through .35 micron filters to ensure digestion to single cells. The cells were then washed with PBS, counted (1-5 million cells/ml) blocked with Fc block from BD as manufacturer's instructions and stained with various antibodies for subsequent flow cytometric analysis in FACS buffer containing 5%FBS and 0.1%NaN3. Gating was done and atleast a 100,000 live cells were enumerated to obtain high numbers of CD45 positive cells as indicated in the figures.
Instrument	BD LSR Fortessa X-20
Software	BD FACS DIVA software was used to collect the data. Analysis was done with FlowJo v10.5
Cell population abundance	CD45 populations were found to be abundantly present as determined by the CD45 antibody staining. Percentages ranging from 20-70%. This population clearly segregated from the other cell populations.
Gating strategy	The cells were first gated by FSC/SSC and doublets were excluded, followed by live/dead staining (Ghost Dye 510, Tonbo). About 60-80% of cells were live. The live cells were then gated for CD45+/FSC and further downstream analysis was done.

☒ Tick this box to confirm that a figure exemplifying the gating strategy is provided in the Supplementary Information.

Magnetic resonance imaging

Experimental design

Design type	*Indicate task or resting state; event-related or block design.*
Design specifications	*Specify the number of blocks, trials or experimental units per session and/or subject, and specify the length of each trial or block (if trials are blocked) and interval between trials.*
Behavioral performance measures	*State number and/or type of variables recorded (e.g. correct button press, response time) and what statistics were used to establish that the subjects were performing the task as expected (e.g. mean, range, and/or standard deviation across subjects).*

Acquisition

Imaging type(s)	*Specify: functional, structural, diffusion, perfusion.*
Field strength	*Specify in Tesla*
Sequence & imaging parameters	*Specify the pulse sequence type (gradient echo, spin echo, etc.), imaging type (EPI, spiral, etc.), field of view, matrix size, slice thickness, orientation and TE/TR/flip angle.*
Area of acquisition	*State whether a whole brain scan was used OR define the area of acquisition, describing how the region was determined.*

Diffusion MRI ☐ Used ☐ Not used

Preprocessing

Preprocessing software	*Provide detail on software version and revision number and on specific parameters (model/functions, brain extraction, segmentation, smoothing kernel size, etc.).*

Normalization	*If data were normalized/standardized, describe the approach(es): specify linear or non-linear and define image types used for transformation OR indicate that data were not normalized and explain rationale for lack of normalization.*
Normalization template	*Describe the template used for normalization/transformation, specifying subject space or group standardized space (e.g. original Talairach, MNI305, ICBM152) OR indicate that the data were not normalized.*
Noise and artifact removal	*Describe your procedure(s) for artifact and structured noise removal, specifying motion parameters, tissue signals and physiological signals (heart rate, respiration).*
Volume censoring	*Define your software and/or method and criteria for volume censoring, and state the extent of such censoring.*

Statistical modeling & inference

Model type and settings	*Specify type (mass univariate, multivariate, RSA, predictive, etc.) and describe essential details of the model at the first and second levels (e.g. fixed, random or mixed effects; drift or auto-correlation).*
Effect(s) tested	*Define precise effect in terms of the task or stimulus conditions instead of psychological concepts and indicate whether ANOVA or factorial designs were used.*

Specify type of analysis: ☐ Whole brain ☐ ROI-based ☐ Both

Statistic type for inference (See Eklund et al. 2016)	*Specify voxel-wise or cluster-wise and report all relevant parameters for cluster-wise methods.*
Correction	*Describe the type of correction and how it is obtained for multiple comparisons (e.g. FWE, FDR, permutation or Monte Carlo).*

Models & analysis

n/a	Involved in the study
☐	☐ Functional and/or effective connectivity
☐	☐ Graph analysis
☐	☐ Multivariate modeling or predictive analysis

Functional and/or effective connectivity	*Report the measures of dependence used and the model details (e.g. Pearson correlation, partial correlation, mutual information).*
Graph analysis	*Report the dependent variable and connectivity measure, specifying weighted graph or binarized graph, subject- or group-level, and the global and/or node summaries used (e.g. clustering coefficient, efficiency, etc.).*
Multivariate modeling and predictive analysis	*Specify independent variables, features extraction and dimension reduction, model, training and evaluation metrics.*

Novus Biologicals antibodies

Total ATM (mouse monoclonal)

Validation

Western Blot: ATM Antibody (2C1) [NB100-309] Mouse monoclonal - HeLa whole cell and nuclear extracts (30 ug) were separated by 5% SDS-PAGE, and the membrane was blotted with ATM antibody (2C1) [NB100-309] diluted at 1:1000.

pATM (mouse monoclonal)

antibodies from Novus: Western Blot: ATM [p Ser1981] Antibody (10H11.E12) [NB100-306], mouse monoclonal - Analysis of ATM-kinase, using ATM [p Ser1981] antibody (10H11.E12) [NB100-306]. Sample: Irradiated or peroxidated human fibroblasts.

YAP1(Rabbit polyclonal)

Genetic Strategies Validation. Knockout Validated: YAP1 Antibody [NB110-58358] - Western lane view shows lysates of HeLa human cervical epithelial carcinoma parental cell line and YAP1 knockout (KO) HeLa cell line. A specific band was detected for YAP1 at approximately 81 kDa (as indicated) using 50 ug/mL of Rabbit Anti-YAP1 Polyclonal Antibody (Catalog # NB110-58358). This experiment was conducted under reducing conditions and using the 12-230 kDa separation system.

TRF2 (Rabbit polyclonal)

Western Blot: TRF-2 Antibody [NB110-57130] - Analysis of HeLa whole cell lysate (A), HeLa nuclear cell lysate (B), k562 cell lysate (C), HepG2 cell lysate (D), NIH/3T3 cell lysate (E), CHO cell lysate (F), PC12 cell lysate (G), and Cos7 cell lysate (H) using antibody at a concentration of 2 ug/ml.

Abcam

YAP1 Y357 validation from Abcam (Rabbit polyclonal)

Lane 1 : Whole cell lysates of HEK-293T cells co-transfected with human YAP1 and human c-Abl
Lane 2 : Whole cell lysates of untransfected HEK-293T cells
Lane 3 : Whole cell lysates of HEK-293T cells co-transfected with human YAP1 and human c-Abl with YAP1 peptide (human 351-362) at 20 µg/ml
Lane 4 : Whole cell lysates of HEK-293T cells co-transfected with human YAP1 and human c-Abl with phospho-YAP1 (human 351-362 [phospho Y357]) immunizing peptide at 20 µg/m

GFP (chicken polyclonal)

ab13970 staining mouse olfactory bulb tissue sections by IHC-Fr.

Sections were PFA fixed, permeabilized in 0.4% Triton-X and blocked with 5% serum for 2 hours at 25°C. The primary antibody was diluted 1/1000 and incubated with the sample for 16 hours at 4°C. An Alexa Fluor® 488 conjugated goat anti-chicken was used as the secondary.

Anti-Interferon gamma antibody (Rabbit polyclonal)

Western blot validation

Cell signaling technologies

c-Abl (Rabbit polyclonal)

Western blot analysis of extracts from various cell lines, using c-Abl Antibody.

NF-kB (Rabbit polyclonal)

Western blot analysis of extracts from various cell lines using NF-κB p65 (D14E12) XP®Rabbit mAb.

Phospho-NF-κB p65 (93H1) Rabbit mAb

Western blot analysis of extracts from HeLa and NIH/3T3 cells, untreated or TNF-α treated (#2169, 20 ng/ml for 5 minutes), using Phospho-NF-κB p65 (Ser536) (93H1) Rabbit mAb (upper) or NF-κB p65 Antibody #3034 (lower).

IκBα (44D4) Rabbit mAb

Western blot analysis of extracts from control HeLa cells (lane 1) or HeLa cells with an apparent in-frame truncation mutation in the gene encoding IκBα (lane 2) using IκBα (44D4) Rabbit mAb, #4812 (upper) or β-actin (13E5) Rabbit mAb #4970 (lower). The change in IκBα molecular weight in the mutated HeLa cells is consistent with an in-frame deletion.

Phospho-IκBα (Ser32) (14D4) Rabbit mAb

Western blot analysis of extracts from HeLa and NIH/3T3 cells, untreated or treated with TNF-α (#2169, 20 ng/ml) for 5 minutes, using Phospho-IκBα (Ser32) (14D4) Rabbit mAb (upper), or IκBα (44D4) Rabbit mAb #4812 (lower).

Histone H2A.X (D17A3) XP® Rabbit mAb

Western blot analysis of extracts from various cell lines using Histone H2A.X (D17A3) XP®Rabbit mAb.

Chk2 (Rabbit polyclonal)

Western blot analysis of extracts from various cell lines using Chk2 Antibody.

pChk2 (Rabbit polyclonal)

Western blot analysis of extracts from Cos cells, untreated or UV-treated (100 mJ/cm^2, 1 hr recovery), using Phospho-Chk2 (Thr68) Antibody

Cleaved Caspase-3 (Asp175) Antibody

Western blot analysis of extracts from HeLa, NIH/3T3 and C6 cells untreated, staurosporine-treated (3hrs, 1 μM in vivo) or cytochrome c-treated (1hr, 0.25 mg/ml in vitro), using Caspase-3 Antibody #9662 (upper) or Cleaved Caspase-3 (Asp175) Antibody (lower).

Millipore Sigma

γH2AX (Anti-p-histone H2A (Ser139) Antibody, clone JBW301 ZooMAb® Mouse Monoclonal)

WB: Untreated (lane 1), and staurosporine-treated (lane 2) Jurkat cell lysates were probed with Anti-phospho-Histone H2A.X (Ser139), clone JBW301, biotin conjugate (0.5µg/ml).

Monoclonal Anti-β-Actin antibody produced in mouse

Cell line lysates were separated on SDS-PAGE and probed with 1:5,000 Monoclonal Anti-β-Actin Clone: AC-15 (Cat. No. A5441). The antibody was developed using Goat Anti-Mouse IgG-Peroxidase (Cat. No. A2304) and a chemiluminescent substrate.

Lanes 1. HeLa, 2. JURKAT, 3. COS7,4. NIH-3T3,5. PC-12,6. RAT2,7. CHO,8. MDBK,9. MDCK

Anti-IL18 antibody (rabbit polyclonal) (Immunohistochemistry)

Staining done for the following tissues, human lung, spleen, lymph node

Adipogen

anti-Caspase-1 (p20) (mouse), mAb (Casper-1)

Mouse caspase-1 (p20) is detected by immunoblotting using anti-Caspase-1 (p20) (mouse), mAb (Casper-1) (Prod. No. AG-20B-0042). Method: Caspase-1 was analyzed by Western blot in cell extracts and supernatants of differentiated bone marrow-derived dendritic cells (BMDCs) from wild-type, NLRP3-/- and caspase-1-/- mice activated or not by 5 µM Nigericin (Prod. No. AG-CN2-0020) for 30 min. Cell extracts and supernatants were separated by SDS-PAGE under reducing conditions, transferred to nitrocellulose and incubated with anti-Caspase-1 (p20) (mouse), mAb (Casper-1) (1µg/ml). Proteins were visualized by a chemiluminescence detection system.

Proteintech

Pro-IL18 detects both pro-form and mature form, mouse monoclonal

WB result of Pre-IL18 antibody (60070-1-Ig, 1:4000) with si-Control and si-Pre-IL18 transfected HeLa cells.

MBL

Mature IL18, mouse monoclonal, clone Clone 39-3F

Monoclonal antibody of 100 µl targeting IL-18 for WB, NT.

Flow cytometric antibodies were used from Biolegend

All the antibodies mentioned in the manuscript as well as the reporting summary from Biolegend were validated by staining splenocytes from mice.
